# Differentiated glioblastoma cells accelerate tumor progression by shaping the tumor microenvironment via CCN1-mediated macrophage infiltration

**DOI:** 10.1186/s40478-021-01124-7

**Published:** 2021-02-22

**Authors:** Atsuhito Uneda, Kazuhiko Kurozumi, Atsushi Fujimura, Kentaro Fujii, Joji Ishida, Yosuke Shimazu, Yoshihiro Otani, Yusuke Tomita, Yasuhiko Hattori, Yuji Matsumoto, Nobushige Tsuboi, Keigo Makino, Shuichiro Hirano, Atsunori Kamiya, Isao Date

**Affiliations:** 1 Department of Neurological Surgery, Okayama University Graduate School of Medicine, Dentistry, and Pharmaceutical Sciences, 2-5-1 Shikata-cho, Kita-ku, Okayama, 700-8558 Japan; 2 Department of Cellular Physiology, Okayama University Graduate School of Medicine, Dentistry and Pharmaceutical Sciences, 2-5-1 Shikata-cho, Kita-ku, Okayama, 700-8558 Japan; 3 Department of Neurosurgery, Hamamatsu University School of Medicine, 1-20-1 Handayama, Higashi-ku, Hamamatsu, Shizuoka 431-3139 Japan; 4 Neutron Therapy Research Center, Okayama University, 2-5-1 Shikata-cho, Kita-ku, Okayama, 700-8558 Japan

**Keywords:** Differentiated glioblastoma cell, Glioblastoma stem cell, CCN1, YAP/TAZ, TEAD, Mesenchymal subtype, Macrophage, Microenvironment, Glioma, Glioblastoma

## Abstract

Glioblastoma (GBM) is the most lethal primary brain tumor characterized by significant cellular heterogeneity, namely tumor cells, including GBM stem-like cells (GSCs) and differentiated GBM cells (DGCs), and non-tumor cells such as endothelial cells, vascular pericytes, macrophages, and other types of immune cells. GSCs are essential to drive tumor progression, whereas the biological roles of DGCs are largely unknown. In this study, we focused on the roles of DGCs in the tumor microenvironment. To this end, we extracted DGC-specific signature genes from transcriptomic profiles of matched pairs of in vitro GSC and DGC models. By evaluating the DGC signature using single cell data, we confirmed the presence of cell subpopulations emulated by in vitro culture models within a primary tumor. The DGC signature was correlated with the mesenchymal subtype and a poor prognosis in large GBM cohorts such as The Cancer Genome Atlas and Ivy Glioblastoma Atlas Project. In silico signaling pathway analysis suggested a role of DGCs in macrophage infiltration. Consistent with in silico findings, in vitro DGC models promoted macrophage migration. In vivo, coimplantation of DGCs and GSCs reduced the survival of tumor xenograft-bearing mice and increased macrophage infiltration into tumor tissue compared with transplantation of GSCs alone. DGCs exhibited a significant increase in YAP/TAZ/TEAD activity compared with GSCs. CCN1, a transcriptional target of YAP/TAZ, was selected from the DGC signature as a candidate secreted protein involved in macrophage recruitment. In fact, CCN1 was secreted abundantly from DGCs, but not GSCs. DGCs promoted macrophage migration in vitro and macrophage infiltration into tumor tissue in vivo through secretion of CCN1. Collectively, these results demonstrate that DGCs contribute to GSC-dependent tumor progression by shaping a mesenchymal microenvironment via CCN1-mediated macrophage infiltration. This study provides new insight into the complex GBM microenvironment consisting of heterogeneous cells.

## Introduction

Glioblastoma (GBM) is the most aggressive and lethal primary brain tumor [33]. Current standard-of-care, including surgery, radiotherapy, and chemotherapy, offers minimal clinical benefits for GBM patients with median survival of less than 16 months [49]. The basis of therapeutic failure is the significant inter- and intra-tumoral heterogeneity of GBM [34, 38, 48, 55, 56]. One aspect of heterogeneity is reflected by the transcriptional subtypes. GBMs have been stratified by bulk gene expression profiles into at least three subtypes, namely proneural, classical, and mesenchymal subtypes [55, 56]. Among these subtypes, the mesenchymal subtype is associated with the worst prognosis and the presence of tumor-associated macrophages/microglia [39, 56].

Another aspect of heterogeneity is reflected by the developmental state of GBM cells in the tumor. In this context, GBM stem-like cells (GSCs) present at the apex of cellular hierarchies and give rise to differentiated GBM cells (DGCs) [1, 23]. GSCs possess capacities for self-renewal, differentiation, and tumor propagation in vivo and exhibit preferential resistance to radiotherapy and chemotherapies [1, 13, 23]. GSCs are essential to drive tumor progression, but the importance of DGCs had been dismissed until a recent study showed that DGCs also contribute to tumor progression in collaboration with GSCs [57]. This study highlighted the potential importance of DGCs in GBM propagation.

The GBM microenvironment consists of heterogeneous cells, namely tumor cells, including GSCs and DGCs, and non-tumor cells including endothelial cells, vascular pericytes, tumor-associated macrophages, and other immune cells [11, 16, 22, 46]. Macrophages are an abundant cellular component of the GBM microenvironment and play multiple roles in GBM progression [15, 22, 46]. Tumor-associated macrophages release several factors, including interleukin (IL)-6 and IL-10, which promote tumor cell growth, facilitate angiogenesis, and suppress the anti-tumor functions of other immune cells [24, 45]. Additionally, GSCs and tumor-associated macrophages interact with each other closely [46]. Tumor-associated macrophages secrete cytokines, such as pleiotrophin and TGF-β1, to maintain the stemness of GSCs and promote invasion of GSCs [24, 46, 47]. GSCs recruit monocyte-derived macrophages from peripheral blood through paracrine periostin and osteopontin signaling [46, 58, 63]. GSCs also promote the survival of M2 tumor-supportive macrophages by secretion of WISP1, which play immune suppressive roles in the tumor microenvironment, [52]. The crosstalk between GSCs and macrophages has been explored actively, but the biological roles of DGCs in GBM progression, especially in the tumor microenvironment, are largely unknown.

Here, using DGC-specific transcriptomic signatures, we investigated the biological roles of DGCs in the tumor microenvironment, and demonstrate that DGCs accelerate GSCs-dependent tumor progression by shaping a mesenchymal microenvironment via CCN1-mediated macrophage infiltration.

## Materials and methods

### Public data acquisition

A deposited RNA sequencing dataset from three matched pairs (MGG4, 6, and 8) of GSCs and DGCs (GSE54791) [51] and single cell RNA-sequencing dataset from four GBM tumors (GSE84465) [16] were downloaded from the NCBI Gene Expression Omnibus (GEO) database. The deposited single cell RNA-sequencing dataset for two-dimensional representation of cellular states are available through the Broad Institute Single-Cell Portal (https://portals.broadinstitute.org/single_cell/study/SCP393/single-cell-rna-seq-of-adult-and-pediatric-glioblastoma) and NCBI GEO GSE131928 [34]. The gene expression data and metadata of The Cancer Genome Atlas (TCGA) GBM (HG-UG133A) and Ivy Glioblastoma Atlas Project (IVY GAP) were downloaded from GlioVis (http://gliovis.bioinfo.cnio.es) [8] or cBioPortal (http://www.cbioportal.org) [10]. The stromal, immune, and tumor purity score of each patient was downloaded from ESTIMATE (http://bioinformatics.mdanderson.org/estimate/) [60].

### Bioinformatic analysis

Unsupervised hierarchical clustering of TCGA GBMs (HG-UG133A) with the DGC signature was performed by Morpheus (https://software.broadinstitute.org/morpheus). Single sample gene-set enrichment analysis (ssGSEA) scores were calculated using the single sample Gene Set Enrichment Analysis Projection (ssGSEAProjection) module in GenePattern (https://cloud.genepattern.org) [2]. Gene set enrichment analysis (GSEA) was performed using the GSEA desktop application (http://software.broadinstitute.org/gsea/downloads.jsp) [50]. To identify specific immune cells linked to DGC signatures or CCN1 in GBM, we examined TCGA GBM (HG-UG133A) dataset for 20 types of immune cells using validated gene set signatures [7, 20]. Gene ontology enrichment analysis (GOEA) was performed through the GlioVis portal. To identify DGC-specific enhancer regions, deposited H3K27ac ChIP-sequencing data from three matched pairs (MGG4, 6, and 8) of GSCs and DGCs (GSE54047) [51] were downloaded from the NCBI Gene Expression Omnibus (GEO) database. DGC-specific enhancer regions were defined by selecting all enhancers present in DGCs, but absent in GSCs using BEDTools [42]. The H3K27ac signals in a 10 kb region of each site were visualized as heatmaps and metaplots using the plotHeatmap and plotProfile functions of deepTools [43]. For *de novo* and known motif enrichment analysis of DGC-specific enhancers, we used the HOMER software package [26]. H3K27ac ChIP-sequencing enrichment plots at the CCN1 locus of three matched GSC and DGC pairs (GSE54047) were visualized using Integrative Genomics Viewer [44].

### Cell culture

The human GBM cell lines were U87ΔEGFR and U251MG provided by Dr. Balveen Kaur (University of Texas Health Science Center, Houston, TX). U87MG cells were purchased from the American Type Culture Collection. A172 and LNZ308 cells were provided by Dr. E. Antonio Chiocca (Brigham and Women’s Hospital, Boston, MA). Patient-derived GBM primary cultures MGG4, MGG8, MGG18, and MGG23 were provided by Dr. Hiroaki Wakimoto (Massachusetts General Hospital, Boston, MA). Normal human astrocytes (NHAs) were purchased from Lonza. U937 monocyte-like cells were purchased from the Japanese Cancer Research Resources Bank. Human embryonic kidney (HEK) 293FT cells were purchased from Thermo Fisher Scientific.

All DGCs, and U251MG, U87MG, U87ΔEGFR, A172, LNZ308, and HEK 293FT cells were cultured in Dulbecco’s modified Eagle’s medium (DMEM) supplemented with 10% fetal bovine serum (FBS), 100 U/ml penicillin, and 100 µg/ml streptomycin. All GSCs were cultured in neurobasal medium (Gibco) supplemented with 0.5 × N2 supplement, 1 × B27 supplement minus vitamin A (Gibco), 0.5 × penicillin/streptomycin/amphotericin B suspension (FUJIFILM Wako), 3 mM L-glutamine (Gibco), 2 μg/ml heparin (Sigma-Aldrich), 20 ng/mL human EGF (PeproTech), and 20 ng/mL human FGF basic (PeproTech). U937 monocyte-like cells were cultured in RPMI 1640 medium supplemented with 10% FBS, 100 U/ml penicillin, and 100 µg/ml streptomycin. NHAs were cultured in AGM BulletKit (Lonza) in accordance with the manufacturer’s instructions. All cells were maintained at 37 °C and 5% CO_2_, and confirmed to be free of mycoplasma. Cell lines were authenticated by Promega using short tandem repeat profiling in December 2016.

### Isolation of GSCs and DGCs by fluorescence-activated cell sorting

MGG4 and MGG8 cells were washed with PBS, blocked with anti-CD16/32 antibodies and normal mouse serum in PBS for 30 min at 4 °C, and then labeled with a Brilliant Violet 421 anti-human CD133 Antibody (BioLegend, #372808). Then, the cells were incubated with propidium iodide, and CD133-positive and -negative cells were sorted by a BD FACSAria III Cell Sorter (Becton Dickinson). A Brilliant Violet 421 Mouse IgG1, κ Isotype Ctrl Antibody (BioLegend, #400158) was used as a negative control to determine the amount non-specific background staining. The sorted CD133-positive cells were cultured in the GSC medium described above. Matched CD133-negative cells were maintained in DMEM supplemented with 10% FBS to maintain their differentiation status. GSC phenotypes were validated by expression of stem cell marker SOX2, their self-renewal capacity (serial neurosphere passaging, in vitro limiting dilution assay), serum-induced cell differentiation, and tumor propagation capacity (in vivo limiting dilution).

### DNA constructs and lentiviral transduction

Lentiviral vectors (LVs) expressing GFP (LV-GFP, Addgene, #26001) or RFP (LV-RFP, Addgene, #25999) were purchased from Addgene. For knockdown experiments, two non-overlapping shRNAs against human CCN1 and TAZ were cloned into pLKO.1 puro (Addgene, #8453). A non-targeting scramble shRNA (#1864, shCONT) and two non-overlapping shRNAs against human YAP (#42540 and #42541) were purchased from Addgene. The target sequences of shRNAs used in this study were as follows. shCCN1-1: CGAACCAGTCAGGTTTACTTA; shCCN1-2 [targeting 3ʹ-untranslated regions (UTRs)]: GGCAGCTATCTGCACTCTAAA; shTAZ-1: GCGATGAATCAGCCTCTGAAT; shTAZ-2: GCGTTCTTGTGACAGATTATA, shYAP-1: GCCACCAAGCTAGATAAAGAA; shYAP-2: CCCAGTTAAATGTTCACCAAT; shCONT: CCTAAGGTTAAGTCGCCCTCG. Lentiviral constructs overexpressing wildtype CCN1 (pTomo-CCN1) or TAZ (pTomo-TAZ) were generated by cloning human CCN1 or TAZ open reading frames, respectively, into the pTomo vector (Addgene, #26291). Site-directed mutagenesis was performed to produce lentiviral constructs expressing D125A, a CCN1 mutant defective for binding αvβ3/αvβ5 integrins, and DM, a CCN1 mutant defective for binding αMβ2/α6β1 integrins [14, 32]. A control lentiviral construct was generated by cloning multiple cloning sites into the pTomo vector. For YAP overexpression, FUW-tetO-wtYAP (Addgene, #84009) and FUdeltaGW-rtTA (Addgene, #19780) were used. For the negative control, FUW-tetO-EGFP (Addgene, #84041) and FUdeltaGW-rtTA were used. HEK 293FT cells were used to generate lentiviral particles by cotransfection of packaging vectors psPAX2 (Addgene, #12260) and pMD2.G (Addgene, #12259) with TransIT-LT1 (Mirus Bio). For lentiviral transduction, cells were transduced with lentiviruses for 48 h and then processed for analyses.

### Mouse intracranial tumor models

All animal experiments were performed with approval from the Committee on the Ethics of Animal Experimentation at Okayama University. For intracranial tumor xenograft models, female BALB/c-nu/nu mice (5–6 weeks old) were purchased from CLEA Japan Inc. The intracranial tumor xenograft models in mice were established as we described previously [28]. Briefly, the mice were anesthetized and tumor cells were stereotactically injected into the right frontal lobe (3 mm lateral and 1 mm anterior from the bregma and 3 mm depth from the dura) using a stereotactic frame (Narishige) and Hamilton syringe (Hamilton). A mouse *de novo* GBM model was generated by stereotactic injection of lentiviruses harboring H-Ras and shP53 [pTomo-HrasV12-IRES-GFP-shp53, the vector plasmid was a kind gift from Dr. Dinorah Friedmann-Morvinski (Tel Aviv University, Tel Aviv, Israel] into the hippocampus of transgenic mice expressing GFAP-Cre, FVB-Tg (GFAP-cre) 25Mes/J (The Jackson Laboratory, #004600) [21]. Mice with neurological deficits or a moribund appearance including a hunched posture, gait changes, lethargy, and weigh loss were sacrificed. Following transcardial perfusion with 4% paraformaldehyde (PFA), brains were harvested, fixed in 4% PFA, and embedded in paraffin or cryopreserved in 30% sucrose for cryosectioning. To observe the effect of macrophage infiltration induced by CCN1, we used U87ΔEGFR, which has rapid tumorigenesis, in a preliminary experiment for the experiment using the GSC-DGC pair.

### Human GBM tissue samples

Fresh GBM tumor tissue for qRT-PCR and immunofluorescence staining were obtained from primary GBM patients who underwent surgical resection at Okayama University Hospital. The study was approved by the ethical committee of the Okayama University Graduate School of Medicine, Dentistry and Pharmaceutical Sciences, Okayama, Japan (approval no. 1608-026). All patients included in the study had provided informed written consent.

### Immunofluorescence staining

Tumor samples from GBM patients and mouse intracranial GBM models were fixed in 4% paraformaldehyde overnight at 4 °C, followed by overnight cryoprotection with 30% sucrose in PBS at 4 °C. Samples were then sectioned at a thickness of 7 µm. Sections were washed with PBS twice, permeabilized, and then blocked with 0.3% Triton X-100, 5% BSA in PBS for 1 h. Then, the sections were stained with primary antibodies against Iba1 (1 µg/mL, FUJIFILM Wako, #019-19741), CD206/MMR (2 µg/mL, R&D Systems, #AF2535), SOX2 (2 µg/mL, R&D Systems, #AF2018), and CCN1/Cyr61 (10 µg/mL, Novus, #NB100-356) overnight at 4 °C, followed by the secondary antibodies against rabbit or goat immunoglobulin G (IgG) labeled with Alexa Fluor dyes (Thermo Fisher Scientific) at room temperature for 1 h. After immunostaining, the samples were mounted with DAPI Fluoromount-G (SouthernBiotech, #0100-20). Images were obtained under an LSM780 confocal laser scanning microscope (Carl Zeiss).

### TEAD luciferase reporter assay

For the TEAD luciferase reporter assay, 1 × 10^5^ DGCs or GSCs were seeded in each well of a 24-well plate. After 12 h, the cells were transfected with the YAP/TAZ-responsive TEAD Firefly luciferase reporter vector 8 × GTIIC-luciferase (Addgene, #34615) (150 ng/cm^2^) and *Renilla* luciferase control reporter vector pGL4.74 [hRluc/TK] (Promega) (100 ng/cm^2^) using TransIT-LT1 (Mirus Bio) in accordance with the manufacturer’s instructions. At 24 h after transfection, Firefly and *Renilla* luciferase activities were quantified using the Dual-Luciferase Reporter Assay System (Promega) in accordance with the manufacturer’s instructions.

### Enzyme-linked immunosorbent assay

Secreted CCN1 protein levels in conditioned media from paired GSCs and DGCs were quantified using an enzyme-linked immunosorbent assay (ELISA). DGCs or GSCs (1 × 10^6^) were seeded in each well of a 12-well plate in DMEM supplemented with 10% FBS or GSC medium, respectively. After 12 h, the media were changed to fresh DMEM without FBS or fresh GSC medium and the cells were cultured for 24 h. At the end point, conditioned media were collected and analyzed using a Human Cyr61/CCN1 ELISA kit (RayBiotech, #ELH-CYR61) in accordance with the manufacturer’s instructions.

### Conditioned medium preparation

Conditioned media (CM) were obtained by culturing GSCs or DGCs at 2 × 10^6^ cells/mL in RPMI 1640 medium without serum for 24 h. The cells were removed by centrifugation at 2000 rpm at 4 °C for 10 min and the CM was sterile filtered through a 0.22-μm filter.

### Migration assay

U937 cells were cultured in RPMI 1640 medium with 10% FBS for 24 h before priming. U937 cells were primed with 5 nM phorbol 12-myristate 13-acetate (Promega, #V1171) for 48 h to become monocyte-derived unpolarized macrophages. Migration assays were performed in 24-well plated with ThinCert cell culture inserts (8-μm pores, Greiner Bio-One) in accordance with the manufacturer’s instructions. Briefly, 5 × 10^5^ primed U937 unpolarized macrophages suspended in serum-free culture medium were seeded in the upper chamber. Medium with recombinant human CCN1 protein (PeproTech, #120-25) or CM was added to the remaining receiver wells. Cells were then allowed to migrate for 48 h before fixation for staining with 0.05% crystal violet (FUJIFILM Wako, #031-04852).

### Western blot analysis

Cells were collected and then lysed in cell lysis buffer (20 mM Tris–HCl, pH 7.5, 150 mM NaCl, 1 mM EDTA, 1 mM Na^2^EGTA, and 0.5% Triton X-100) containing a cOmplete Protease Inhibitor Cocktail (Sigma-Aldrich) and PhosSTOP phosphatase inhibitor cocktail (Sigma-Aldrich). After sonication, lysates were centrifuged at 15,000 rpm at 4 °C, for 10 min. The protein concentration of the supernatants was measured using a bicinchoninic acid protein assay (Thermo Fisher Scientific). The supernatants were added to a 1/3 volume of 4 × SDS sample buffer (240 mM Tris–HCl, pH 6.8, 8% SDS, 40% glycerol, 0.1% bromophenol blue, and 20% 2-mercaptoethanol) and boiled at 95 °C for 5 min. Equal amounts of protein samples were applied to SDS-PAGE and then transferred to a PVDF membrane (Immobilon-P, 0.45 μm) (MilliporeSigma). The membrane was blocked with 0.5% dry skim milk in TBST. After blocking, the membranes were incubated with primary antibodies overnight at 4 °C and then with secondary antibodies for 1 h at room temperature. The signals were developed with Clarity Western ECL Substrate (Bio-Rad Laboratories) and detected with a ChemiDoc imaging system (Bio-Rad Laboratories). The primary antibodies were anti-CCN1/CYR61(1:1000, Cell Signaling Technology, #14479), anti-YAP (1:1000, Santa Cruz Biotechnology, #sc-101199), anti-TAZ/WWTR1(1:1000, Sigma-Aldrich, #HPA007415), anti-SOX2 ((2 µg/mL, R&D Systems, #AF2018), and anti-GAPDH (1:1000, Sigma-Aldrich, #MAB374). The secondary antibodies were horseradish peroxidase-conjugated anti-mouse IgG (1:4,000, Cell Signaling Technology, #7076), anti-rabbit IgG (1:4,000, Cell Signaling Technology, #7074), and anti-goat IgG (1:4,000, Sigma-Aldrich, #A5420).

### Quantitative RT-PCR

Trizol reagent (Invitrogen) was used to isolate total cellular RNA from cell pellets. After digestion of genomic DNA using Recombinant DNase I (Takara Bio Inc.), a PrimeScript RT reagent Kit (Takara Bio Inc.) was used for reverse transcription into cDNA. Quantitative real-time PCR was performed with a Rotor-Gene Q (QIAGEN) using Luna Universal qPCR Master Mix (New England Biolabs). qPCR primers used in this study were as follows. human CCN1 forward 5ʹ-CCTTGTGGACAGCCAGTGTA-3ʹ and reverse 5ʹ-ACTTGGGCCGGTATTTCTTC-3ʹ; human YAP forward 5ʹ-TAGCCCTGCGTAGCCAGTTA-3ʹ and reverse 5ʹ-TCATGCTTAGTCCACTGTCTGT-3ʹ; human TAZ forward 5ʹ-TCCCAGCCAAATCTCGTGATG-3ʹ and reverse 5ʹ-AGCGCATTGGGCATACTCAT-3ʹ; 18S RNA forward 5ʹ-GTAACCCGTTGAACCCCATT-3ʹ and reverse 5ʹ-CCATCCAATCGGTAGTAGCG-3ʹ.

### Statistical analysis

GraphPad Prism 8 software was used to conduct statistical analysis of all data. Data are represented as the mean and SEM. Kaplan–Meier survival curves were generated using GraphPad Prism 8 software and the log-rank test was performed to assess statistical significances between groups. The Student’s t-test was used for comparisons between two groups. Comparisons between multiple groups were performed with one-way ANOVA with Tukey’s multiple comparisons test. Pearson’s correlation test was used to measure the strength of the association between two variables. The chi-squared test was performed to examine differences between categorical variables. *P* values were designated as *P < 0.05, **P < 0.01 ***P < 0.001, ****P < 0.0001, and ns non-significant (*P* > 0.05).

## Results

### Determination of DGC-specific transcriptomic signatures

**Fig. 1:**
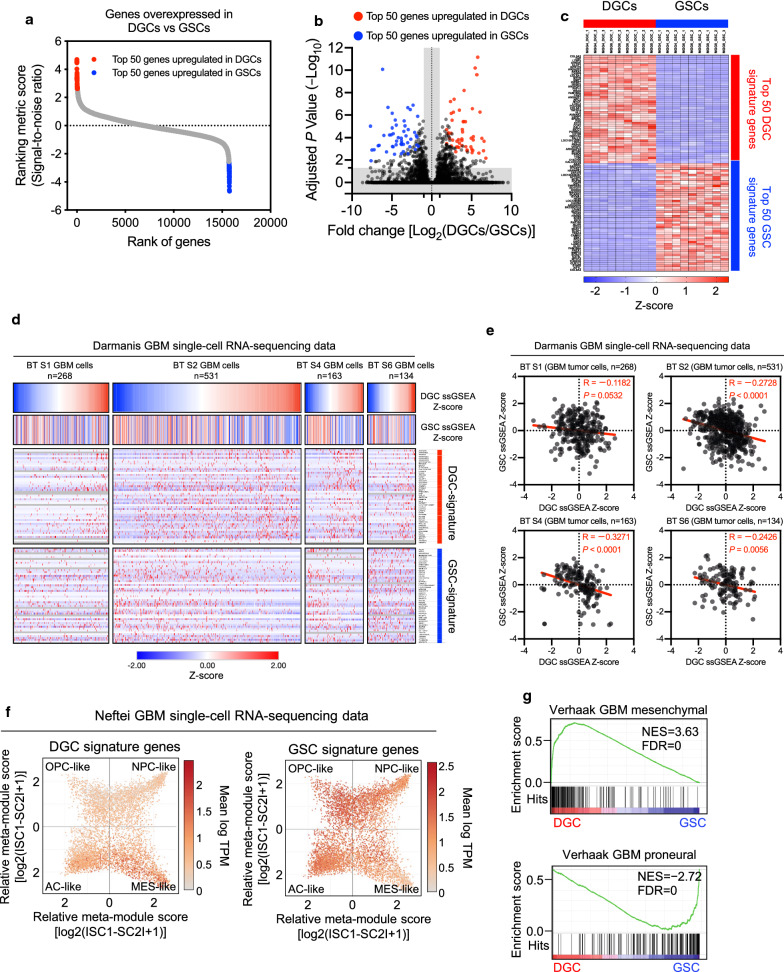
Determination of DGC-specific transcriptomic signatures and their validation in single cell RNA-sequencing data. **a** Dot plot showing the ranking metric score (signal-to-noise ratio) of DGCs versus GSCs. Red and blue dots indicate the top 50 significantly altered genes in DGCs and GDCs. On the x axis, genes farther to the left have higher expression in DGCs, whereas genes farther to the right have higher expression in GSCs. NCBI Gene Expression Omnibus GSE54791. **b** Volcano plot comparing gene expression between DGCs and GSCs. Each dot represents one gene. Red and blue dots indicate the top 50 significantly altered genes in DGCs and GDCs. Genes were considered to be significantly different when the adjusted *P* value was < 0.05 and the difference of the mean fold change was > 2. NCBI Gene Expression Omnibus GSE54791. **c** Heat map of the top 50 genes exclusively upregulated in DGCs and GSCs. NCBI Gene Expression Omnibus GSE54791. **d** Heat map shows expression of DGC and GSC signature ssGSEA scores and genes of each signature (rows) in individual GBM cells (columns) of single cell RNA-sequencing data from four GBM tumors (BT S1, BT S2, BT S4, and BT S6). Cells were grouped by the tumor and ordered by the DGC signature ssGSEA score. NCBI Gene Expression Omnibus GSE84465. **e** Correlation analysis between DGC and GSC signature ssGSEA scores in single cell RNA-sequencing data from four GBM tumors (BT S1, BT S2, BT S4, and BT S6). Pearson’s correlation test. NCBI Gene Expression Omnibus GSE84465. **f** Expression of DGC signature genes (left) and GSC signature genes (right) in a cluster of two-dimensional representation of cellular states. Each quadrant corresponds to one cellular state, the exact positions of malignant cells (dots) reflect their relative scores for the meta-modules, and their colors reflect gene expression levels. AC, astrocyte, MES, mesenchymal, OPC, oligodendrocyte-progenitor-cell, NPC, neural-progenitor-cell, TPM, transcripts per million. Source data are available through the Broad Institute Single-Cell Portal. (https://portals.broadinstitute.org/single_cell/study/SCP393/single-cell-rna-seq-of-adult-and-pediatric-glioblastoma) and NCBI Gene Expression Omnibus GSE131928. **g** GSEA analysis of mesenchymal and proneural subtypes of DGCs compared with GSCs. NCBI Gene Expression Omnibus GSE54791. NES: normalized enrichment score. FDR: false discovery rate

To investigate transcriptomic profiles of DGCs compared with GSCs, we analyzed a deposited RNA sequencing dataset from three matched pairs of GSCs and DGCs [51] and extracted the top 50 genes that were differentially expressed between DGCs and GSCs (DGC and GSC signature genes) on the basis of the ranking metric score (signal-to-noise ratio) (Fig. 1a–c, Additional file 1: S1a,b).

### Validation of DGC-specific transcriptomic signatures in single cell RNA-sequencing data

Next, to confirm the presence of cell subpopulations emulated by in vitro DGC and GSC culture models within a primary tumor, we evaluated the DGC and GSC signatures in the deposited single cell RNA-sequencing dataset from four GBM tumors [16]. GBM cells aligned with differentiation and stemness gradients in each tumor (Fig. 1d). Negative correlations between DGC and GSC signature single sample gene set enrichment analysis (ssGSEA) scores [2] were also confirmed in the single cell RNA-sequencing data (Fig. 1e), which was consistent with the findings observed in the RNA sequencing dataset of in vitro culture models. A recent study has demonstrated that malignant cells in GBM exist in four main cellular states that recapitulate neural-progenitor-like (NPC-like), oligodendrocyte-progenitor-like (OPC-like), astrocyte-like (AC-like), and mesenchymal-like (MES-like) states [34]. Thus, we assessed the expression of DGC and GSC signature genes across the four GBM cellular states. The results showed that DGCs were enriched in the MES-like state, while GSCs were enriched in OPC-, NPC-, and AC-like states (Fig. 1f). Gene set enrichment analysis (GSEA) [50] of the in vitro culture models showed significant enrichment of the mesenchymal gene set in DGCs and the proneural gene set in GSCs (Fig. 1g). These results suggest that GBM tumors contain cell subpopulations modeled by the GSC and DGC culture models.

### Validation of DGC-specific transcriptomic signatures in larger tumor cohorts

**Fig. 2:**
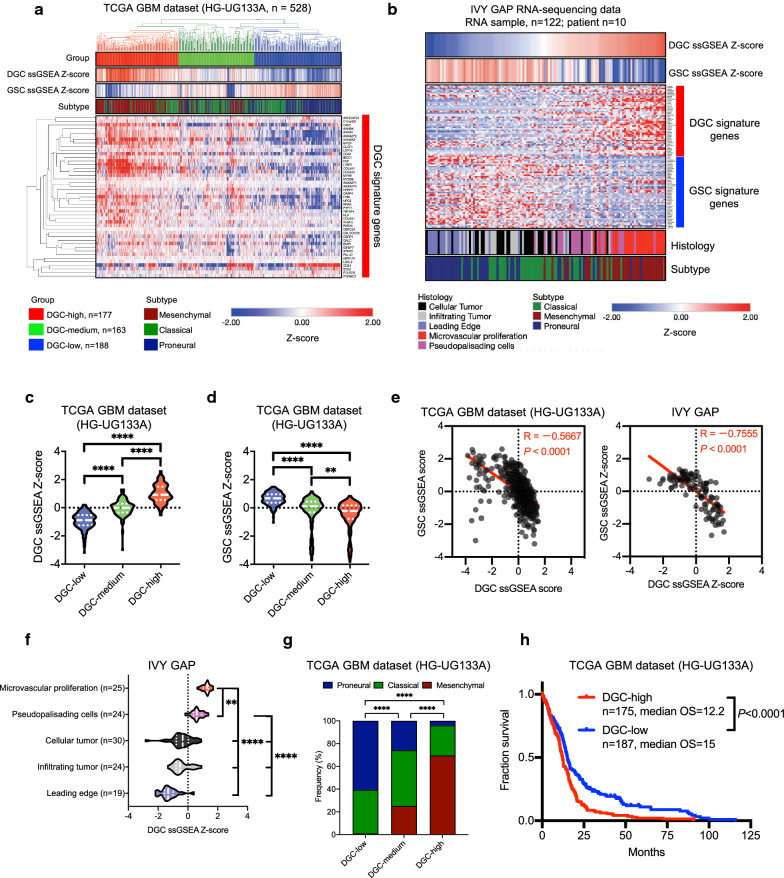
Validation of DGC-specific transcriptomic signatures in larger tumor cohorts and their correlation with the mesenchymal subtype and a poor patient prognosis. **a** Hierarchical clustering of human TCGA GBM samples (HG-UG133A, n = 528) into DGC-high (n = 177), DGC-medium (n = 163), and DGC-low (n = 188) groups using DGC signature genes. The corresponding DGC signature groups for each sample determined via hierarchical clustering are labeled (top). Heat map showing Z-scores of DGC and GSC signatures determined by single sample gene set enrichment analysis (ssGSEA) of individual GBM samples (second and third from top). The corresponding GBM subtype for each sample is labeled (fourth from top). **b** Heat map shows expression the DGC and GSC signature ssGSEA scores (top and second from top) and genes of each signature (rows) for each RNA sample in the anatomic structure study dataset (122 RNA sample data from 10 patients) from the IVY GAP database (columns). The corresponding GBM subtype and histology for each sample is are (bottom and second from bottom). **c** ssGSEA scores of DGC signature genes of DGC-high (n = 177), DGC-medium (n = 163), and DGC-low (n = 188) patients in TCGA GBM dataset (HG-UG133A, n = 528). Violin plots represent the median (thick dotted line) and quartiles (dotted line). ****P < 0.0001, one-way ANOVA with Tukey’s multiple comparisons test. **d** ssGSEA scores of the GSC signature genes of three DGC groups in TCGA GBM dataset (HG-UG133A, n = 528). Violin plots represent the median (thick dotted line) and quartiles (dotted line). **P < 0.01, ****P < 0.0001, one-way ANOVA with Tukey’s multiple comparisons test. **e** Correlation analysis between DGC and GSC ssGSEA scores in TCGA GBM dataset (HG-UG133A, n = 528) and IVY GAP dataset (122 RNA sample data from 10 patients). Pearson’s correlation test. **f** ssGSEA scores of the DGC signature genes in multiple regions of the IVY GAP data (122 RNA sample data from 10 patients). Violin plots represent the median (thick dotted line) and quartiles (dotted line). **P < 0.01, ****P < 0.0001, one-way ANOVA with Tukey’s multiple comparisons test. **g** Molecular subtype distribution among DGC signature groups in TCGA GBM dataset (HG-UG133A, n = 528). ****P < 0.0001, chi-squared test. **h** Kaplan–Meier analyses between patients in DGC-high and DGC-low groups of TCGA GBM dataset (HG-UG133A). Log-rank *P* value analyses

To determine the validity of the DGC and GSC transcriptomic signatures in larger tumor cohorts, we examined The Cancer Genome Atlas (TCGA) GBM dataset (HG-UG133A), which contains data from 528 GBMs (Fig. 2a), and the Ivy Glioblastoma Atlas Project (IVY GAP) database, that contains 122 RNA sample data from 10 patients (Fig. 2b). We performed unsupervised clustering of TCGA GBM dataset with the DGC signature to generate three groups: DGC-high (n = 177), DGC-medium (n = 163), and DGC-low (n = 188) (Fig. 2a). To assess the robustness of our clustering, we calculated ssGSEA scores [2] of the DGC and GSC signatures for individual GBM samples. The ssGSEA scores of the DGC signature were highly enriched in the DGC-high group compared with the DGC-low group (Fig. 2c). However, the ssGSEA scores of the GSC signature were highly enriched in the DGC-low group compared with the DGC-high group (Fig. 2d). The negative correlation between DGC and GSC signatures was confirmed in TCGA GBM and IVY GAP datasets (Fig. 2e). These results suggested the validity of the application of DGC signatures to clinical cohorts and our clustering shown in Fig. 2a.

### Transcriptomic DGC signatures correlate with the mesenchymal subtype and poor patient prognoses

We next investigated the clinical and anatomical relevances of the DGC signature in GBM cohorts. Anatomically, regions of microvascular proliferation and pseudopalisading cells expressed the DGC signature more in GBM tissues, whereas the leading edge and infiltrating tumor regions expressed the GSC signature more (Fig. 2b, f, Additional file 1: S2a). Consistent with the findings shown in Fig. 1g, the DGC signature was associated with the mesenchymal subtype in TCGA GBM and IVY GAP datasets (Fig. 2g, Additional file 1: S2b). Furthermore, patients with higher expression of the DGC signature exhibited poorer survival when grouped by both the clustering shown in Fig. 2a and ssGSEA score (Fig. 2h, Additional file 1: S2c). These results suggest that transcriptomic DGC signatures correlate with the mesenchymal subtype and poor patient prognoses.

### Transcriptomic DGC signatures are associated with immune responses and macrophage signatures

**Fig. 3:**
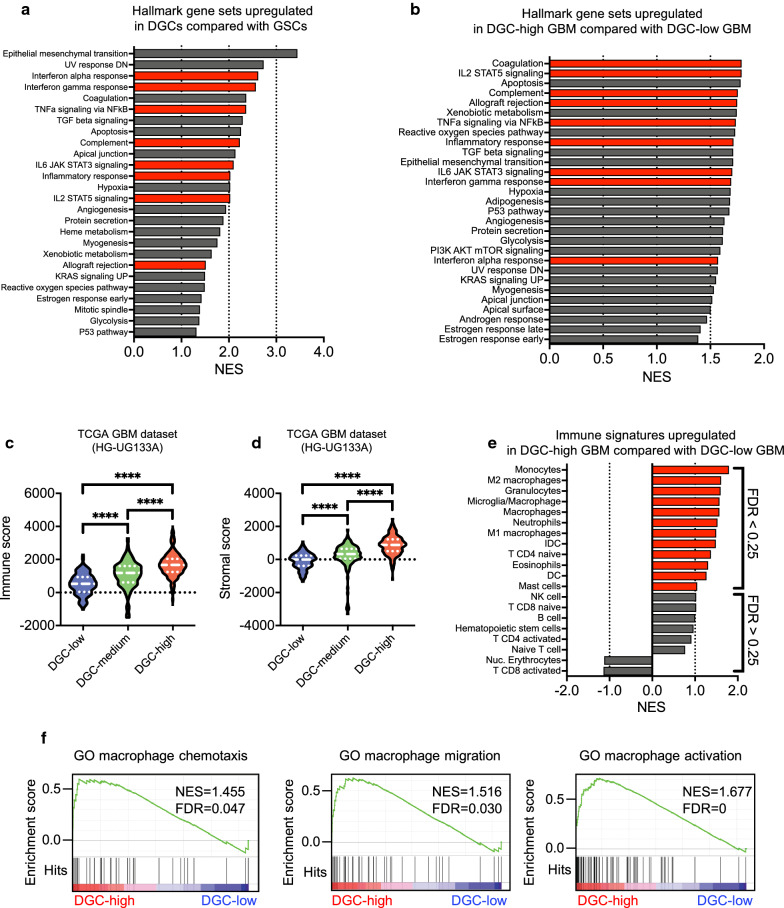
Transcriptomic DGC signatures are associated with immune responses and macrophage signatures. **a** Bar graph showing the normalized enrichment score (NES) of GSEA analysis of hallmark gene sets upregulated in DGCs compared with GSCs. Twenty-six gene sets were significantly enriched at a false discovery rate (FDR) of < 0.25 and nominal *P* value of < 0.05 in DGCs. NCBI Gene Expression Omnibus GSE54791. Red bars indicate signatures related to immune responses. **b** Bar graph showing the NES of GSEA analysis of hallmark gene sets upregulated in DGC-high GBMs (n = 177) compared with DGC-low GBMs (n = 188). Twenty-nine gene sets were significantly enriched at FDR < 0.25 and nominal *P* value < 0.05 in DGC-high GBMs. TCGA GBM dataset (HG-UG133A, n = 528). Red bars indicate signatures related to immune responses. **c** Immune scores of DGC-high (n = 177), DGC-medium (n = 163), and DGC-low (n = 188) patients in TCGA GBM dataset (HG-UG133A, n = 528). Violin plots represent the median (thick dotted line) and quartiles (dotted line). ****P < 0.0001, one-way ANOVA with Tukey’s multiple comparisons test. **d** Stromal scores of DGC-high, -medium, and -low patients in TCGA GBM dataset (HG-UG133A). Violin plots represent the median (thick dotted line) and quartiles (dotted line). ****P < 0.0001, one-way ANOVA with Tukey’s multiple comparisons test. **e** GSEA analysis of various types of immune cell signatures upregulated in DGC-high (n = 177) compared with DGC-low (n = 188) patients in TCGA GBM dataset (HG-UG133A). Red bars indicate FDR < 0.25. **f** GSEA analysis of macrophage-related signatures upregulated in DGC-high (n = 177) compared with DGC-low (n = 188) patients in TCGA GBM dataset (HG-UG133A)

To investigate the biological role of DGCs in GBM, we explored signaling pathways correlated with DGCs by GSEA using transcriptomic data of the in vitro models (matched pairs of GSCs and DGCs) and TCGA GBM cohorts (Fig. 3a, b). GSEA of hallmark gene sets revealed prominent representation of immune response gene sets in the in vitro DGC models and DGC-high GBM, including the interferon α/γ response, TNF α/NF-κB signaling, inflammatory response, interleukin-2 (IL-2)/STAT5 signaling, and IL-6/STAT3 signaling (Fig. 3a, b, Additional file 1: S3a).

Next, to predict the presence of stromal/immune cell populations in tumors and tumor purity, we used the ESTIMATE method [60]. Using TCGA GBM dataset, we found that the DGC-high GBM group, which was enriched in the mesenchymal subtype (Fig. 2g), exhibited high stromal and immune signatures and low tumor purity (Fig. 3c, d, Additional file 1: S3b). These results were consistent with previous findings of an increased presence of stromal and immune cells in mesenchymal type GBM [56].

To identify specific immune cells linked to DGC signatures, we examined TCGA GBM dataset for various types of immune cells using validated gene set signatures [7, 20]. Analysis of immune cell signatures demonstrated that high DGC-signature expression correlated with significant enrichment of macrophages (total, M1, and M2-macrophages), microglia, and monocytes (Fig. 3e). Therefore, we assessed macrophage-related gene sets (macrophage chemoattractant, migration, and activation) by GSEA and found that the DGC-high GBM group exhibited significant enrichment of these gene sets (Fig. 3f). Taken together, these in silico findings suggest a role of DGCs in macrophage infiltration into GBM.

### DGCs promote macrophage infiltration and tumor progression in cooperation with GSCs

**Fig. 4:**
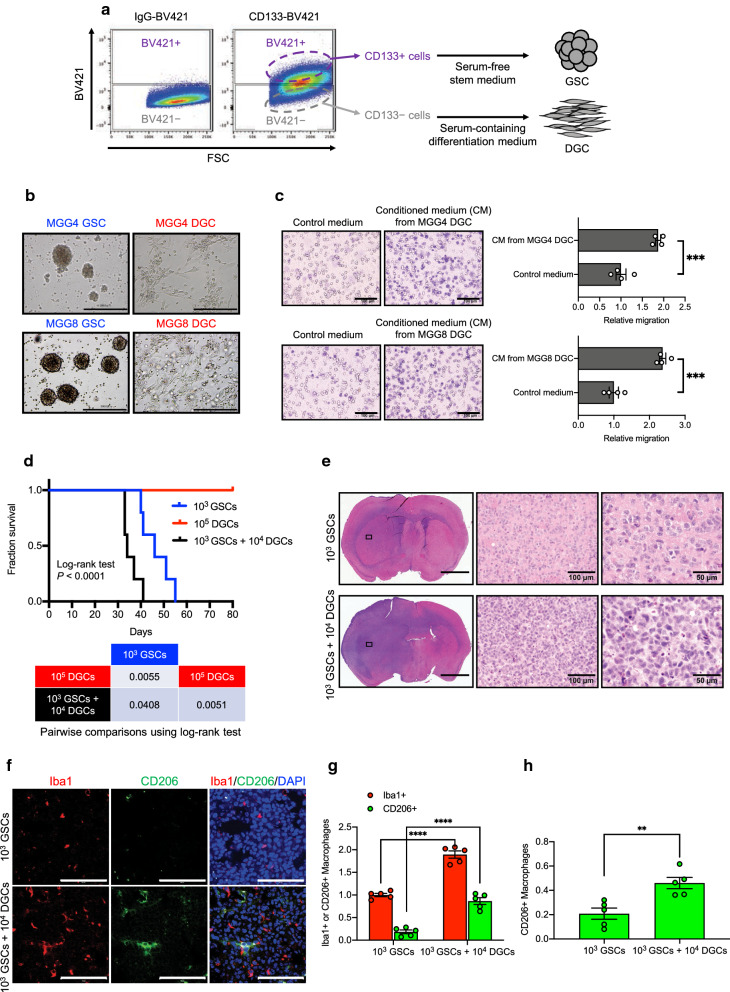
DGCs promote macrophage infiltration and tumor progression in cooperation with GSCs. **a** Flow cytometric analysis of CD133 in MGG8 cells. Sorted CD133 + cells were cultured as GSCs and CD133-cells were cultured as DGCs. **b** Representative images of paired GSCs and DGCs derived from two primary human GBM specimens (MGG4 and MGG8). Scale bar, 300 µm. **c** Representative image (left panel) and quantification (right panel) of transwell analysis of U937 macrophages upon stimulation with control medium or conditioned medium (CM) from DGCs (MGG4 and MGG8). Scale bar, 100 μm. n = 4 biological replicates, mean ± SEM, ***P < 0.001, Student’s t-test. **d** Kaplan–Meier (upper) and log-rank *P* value (bottom) analyses of mice bearing orthotopic xenografts of 1 × 10^3^ glioma stem-like cells (GSCs) alone, 1 × 10^5^ differentiated glioblastoma cells (DGCs) alone, or cotransplanted 1 × 10^4^ DGCs with 1 × 10^3^ GSCs derived from MGG8 cells. **e** Representative H&E stainings of tumor-bearing brains harvested at 30 days after implantation of 1 × 10^3^ GSCs alone, or 1 × 10^3^ GSCs plus 1 × 10^4^ matched DGCs derived from MGG8. Scale bars indicate 2000, 100 and 50 µm in gross and detail views, respectively. Middle and right panels are high magnifications of the areas marked by rectangles in left panels. **f** Representative confocal images of tumor-bearing brains harvested at 30 days after implantation of 1 × 10^3^ GSCs alone, or 1 × 10^3^ GSCs plus 1 × 10^4^ matched DGCs derived from MGG8. Scale bar, 100 µm. Iba1 (red), CD206 (green), and DAPI (blue). **g** Quantitation of pan-macrophage (Iba1^+^) and M2 macrophage (CD206^+^) densities in xenografts formed by 1 × 10^3^ GSCs alone or 1 × 10^3^ GSCs plus 1 × 10^4^ matched DGCs derived from MGG8 cells. The total number of macrophages was counted in five randomly selected fields per sample. n = 5 biological replicates, mean ± SEM, ***P < 0.001, ****P < 0.0001, Student’s t-test. **h** Quantitation of the fraction of M2 macrophages (CD206^+^). The fraction was determined by M2 macrophages (CD206^+^) among pan-macrophages (Iba1^+^) in 1 × 10^3^ GSCs alone or 1 × 10^3^ GSCs plus 1 × 10^3^ matched DGCs xenografts. The total number of macrophages was counted in five randomly selected fields per sample. n = 5 biological replicates. Data are represented as means ± SEM. **P < 0.01, Student’s t-test

To perform matched GSC and DGC experiments, we adopted an established protocol to isolate GSCs and DGCs. GBM tumor cells were isolated by fluorescence-activated cell sorting on the basis of CD133, a stem-cell marker [1, 51, 57]. The CD133-positive cells were cultured as GSCs in serum-free stem cell medium and CD133-negative cells were cultured as DGCs in serum-containing differentiation medium (Fig. 4a). GSCs grew as spheres under serum-free conditions and DGCs expanded as adherent monolayers under serum-containing conditions (Fig. 4b). Consistent with the in silico findings in Fig. 3, conditioned medium (CM) of DGCs isolated from patient-derived GBM cells (MGG4 and MGG8) and human GBM cell line U87ΔEGFR exhibited an increase in U937 macrophage migration relative to the control medium in transwell migration assays (Fig. 4c, Additional file 1: S4a). To confirm the contribution of DGCs to tumor progression, we implanted GSCs alone, matched DGCs alone, or their combination derived from MGG8 cells into the brains of immunocompromised mice. We determined the ratio of DGCs and GSCs by referring to a previous study [57]. As reported in previous studies [51, 57], coimplantation of DGCs and GSCs reduced the survival of tumor xenograft-bearing mice compared with GSCs alone, whereas as many as 1 × 10^5^ DGCs alone did not initiate a tumor (Fig. 4d). Hematoxylin and eosin (H&E) staining of whole brain sections showed increases in the tumor size and cell density with coimplantation of GSCs and DGCs (Fig. 4e). Furthermore, coimplantation of DGCs and GSCs increased infiltration of CD206-positive tumor-supportive macrophages (M2 macrophages) compared with GSCs alone (Fig. 4f–h). Finally, to confirm successful implantation of DGCs into the brains of recipient mice only when cotransplanted with GSCs, we adopted tracing strategies with tumor cells stably expressing fluorescent proteins (Additional file 1: Fig. S4b). To this end, GSCs and DGCs were infected with lentiviral vectors (LVs) expressing GFP or RFP (Additional file 1: Fig. S4c). We implanted GSCs labeled with RFP alone, DGCs labeled with GFP alone, or their combination (Mixed) into the brains of recipient mice. DGCs were successfully engrafted in mouse brains when cotransplanted (Mixed) with GSCs, but transplantation of DGCs alone did not initiate a tumor (Additional file 1: Fig. S4d). Collectively, these results demonstrate that DGCs promote macrophage infiltration and tumor progression in cooperation with GSCs.

### Identification of DGC-specific transcriptional regulators

**Fig. 5:**
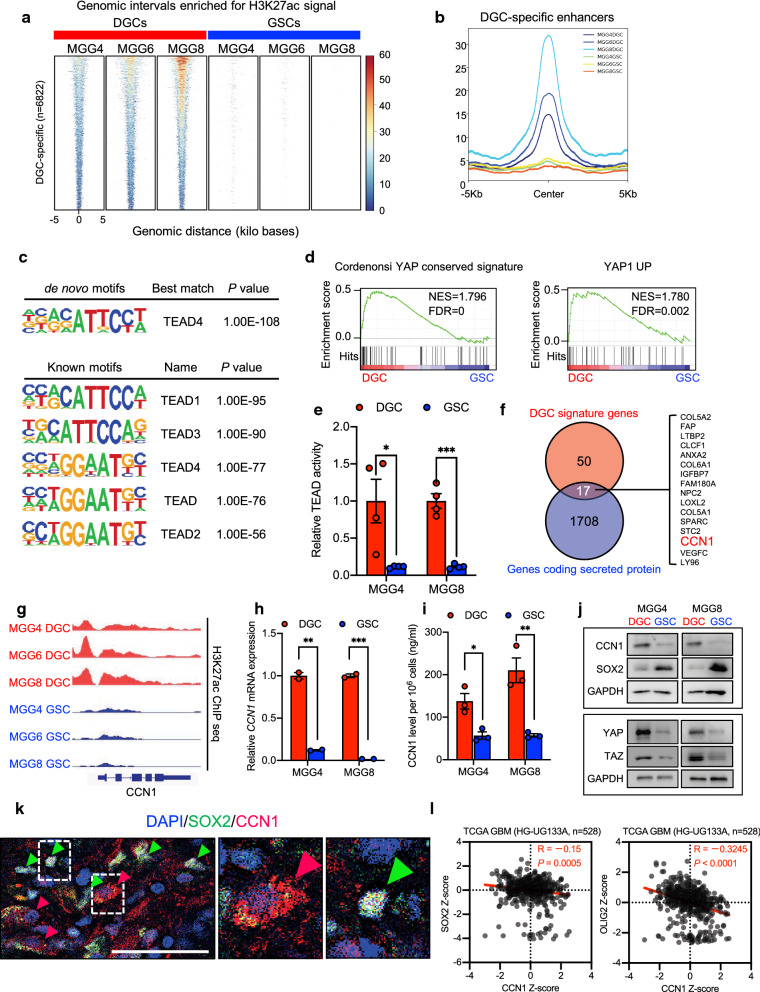
Identification of DGC-specific transcriptional regulators and a potential protein that governs macrophage recruitment by DGCs. **a** Heat map showing H3K27ac signals of all DGC-specific enhancers in three matched GSCs and DGCs (MGG4, MGG6, and MGG8). H3K27ac chip sequencing data were derived from NCBI Gene Expression Omnibus GSE54047. **b** Metaplot showing average H3K27ac signals of all DGC-specific enhancers in three matched GSCs and DGCs (MGG4, MGG6, and MGG8). H3K27ac chip sequencing data were derived from NCBI Gene Expression Omnibus GSE54047. **c** *De novo* and known motif enrichment analysis of DGC-specific enhancers defined in **a**, **b** displayed enrichment for transcriptional motifs of the TEAD transcription factor family. **d** GSEA analysis of YAP-related signatures upregulated in DGCs compared with GSCs. NCBI Gene Expression Omnibus GSE54791. NES: normalized enrichment score. FDR: false discovery rate. **e** Quantification of the TEAD luciferase reporter assay of matched pairs of GSCs and DGCs derived from MGG4 and MGG8 cells. Data are presented as the mean ± SEM of four independent experiments. *P < 0.05, ***P < 0.001. **f** Venn diagram showing the intersection between DGC signature genes (n = 50) and genes encoding secreted proteins from the human protein atlas (n = 1708). CCN1 was selected because it is a target gene of the YAP/TAZ-TEAD complex. **g** H3K27ac ChIP-sequencing enrichment plot at the CCN1 locus of three matched pairs of GSCs and DGCs (MGG4, MGG6, and MGG8). Matched pairs of GSC and DGC data were derived from NCBI Gene Expression Omnibus GSE54047. **h** qRT-PCR quantification of CCN1 mRNA levels in matched pairs of GSCs and DGCs derived from MGG4 and MGG8 cells. Data are presented as the mean ± SEM of two independent experiments. **P < 0.01, ***P < 0.001. **i** ELISA quantification of secreted CCN1 protein levels in conditioned media from paired GSCs and DGCs derived from MGG4 and MGG8 cells. Data are presented as the mean ± SEM of three independent experiments. **P < 0.01. **j** DGCs express elevated CCN1, YAP and TAZ protein levels relative to GSCs. Protein levels of CCN1, YAP, TAZ and SOX2 (GSC marker) were assessed by immunoblotting in two pairs of GSCs and DGCs of patient-derived glioma cell lines (MGG4 and MGG8). GAPDH was used as a loading control. **k** Immunofluorescence staining of SOX2 (green) and CCN1 (red) in frozen sections of human GBM specimens counterstained with DAPI (blue). Scale bar, 50 μm. Green arrowheads, SOX2-positive GSC-like cells. Red arrowheads, SOX2-negative DGC-like cells. Middle and right panels are high magnifications of the rectangle area in the left panel. **l** Correlation between CCN1, SOX2, and OLIG2 in TCGA GBM (HG-U133A) dataset. Red numbers indicate the correlation R-value. Pearson’s correlation test

To identify DGC-specific enhancers, we compared the epigenetic landscape of three matched pairs (MGG4, 6, and 8) of GSCs and DGCs by analysis of H3K27ac ChIP-sequencing data (Fig. 5a, b) [51]. DGC-specific enhancers displayed enrichment for transcriptional motifs of TEA domain family member (TEAD) 1–4 and activator protein-1 (AP-1) (Fig. 5c, Additional file 1: S5a). AP-1 is a dimer of JUN (JUN, JUNB, and JUND) and FOS (FOS, FOSB, FOSL1/FRA1, and FOSL2/FRA2) families of leucine-zipper proteins [18]. Transcriptional coactivator with PDZ-binding motif (TAZ), also known by gene name WW domain-containing transcription regulator 1 (WWTR1), and its paralog, Yes-associated protein (YAP), also known by gene name YAP1, are the two nuclear effectors of the Hippo signaling pathway. YAP/TAZ/TEAD and AP-1 form a complex that synergistically activates YAP/TAZ target genes [62]. Indeed, TEAD transcriptional activity was regulated by YAP/TAZ (Additional file 1: Fig. S5b–e). Furthermore, DGCs exhibited significant enrichment of YAP-related signatures (Fig. 5d) and upregulation of TEAD transcriptional activity (Fig. 5e) compared with GSCs. These findings were consistent with the results of motif enrichment analysis. Collectively, these results demonstrated that DGCs exhibited a significant increase in YAP/TAZ/TEAD activity.

### CCN1 is a potential protein secreted from DGCs to regulate macrophage recruitment

To identify the DGC signature genes that governed macrophage recruitment for further study, we selected 17 genes encoding secreted proteins (The Human Protein Atlas [54]: https://www.proteinatlas.org/humanproteome/tissue/secretome) from the 50 DGC signature genes (Fig. 5f) because macrophages are recruited by secreted factors. Of these 17 genes, cellular communication network factor 1 (CCN1), also known as cysteine rich angiogenic inducer 61 (CYR61), was selected for validation and further analyses (Fig. 5f) because it is a target gene of the YAP/TAZ/TEAD complex [40]. In fact, protein and mRNA levels of CCN1 were positively correlated to YAP (YAP1) and TAZ (WWTR1) in GBM cells and clinical samples from our institution and TCGA (Additional file 1: Fig. S5f–h). Expression of CCN1 was regulated by YAP/TAZ (Additional file 1: Fig. S5i–l).

To directly validate upregulation of CCN1 in DGCs, we examined the active enhancer landscape of CCN1 across three matched pairs of GSCs and DGCs derived from GBM patients [51], which revealed markedly active CCN1 enhancers in DGCs as measured by H3K27ac peak levels (Fig. 5g). Next, we quantified the relative expression levels of CCN1 by qRT-PCR between DGCs and GSCs (MGG4 and MGG8), which demonstrated the increased expression levels of CCN1 in DGCs (Fig. 5h). To confirm translation of these mRNAs into proteins, we measured CCN1 protein by the enzyme-linked immunosorbent assay (ELISA), which confirmed that DGCs secreted higher levels of CCN1 than GSCs (Fig. 5i). Furthermore, western blotting showed increases in CCN1 and YAP/TAZ proteins in DGCs compared with those in GSCs (Fig. 5j). To exclude the effects of cell culture conditions on the higher expression of CCN1 in DGCs, we performed dual immunofluorescence staining of GBM surgical specimens for CCN1 and GSC marker SOX2. In human GBM specimens, DGC-like cells (SOX2 negative and CCN1 positive) and GSC-like cells (SOX2 positive and CCN1 negative) were observed (Fig. 5k). Expression of CCN1 mRNA was negatively correlated to GSC markers SOX2 and OLIG2 in clinical samples of TCGA (Fig. 5l). Taken together, these findings support that CCN1 as a potential protein secreted from DGCs, which governs macrophage recruitment.

### DGCs augments macrophage infiltration into GBM through secretion of CCN1

**Fig. 6:**
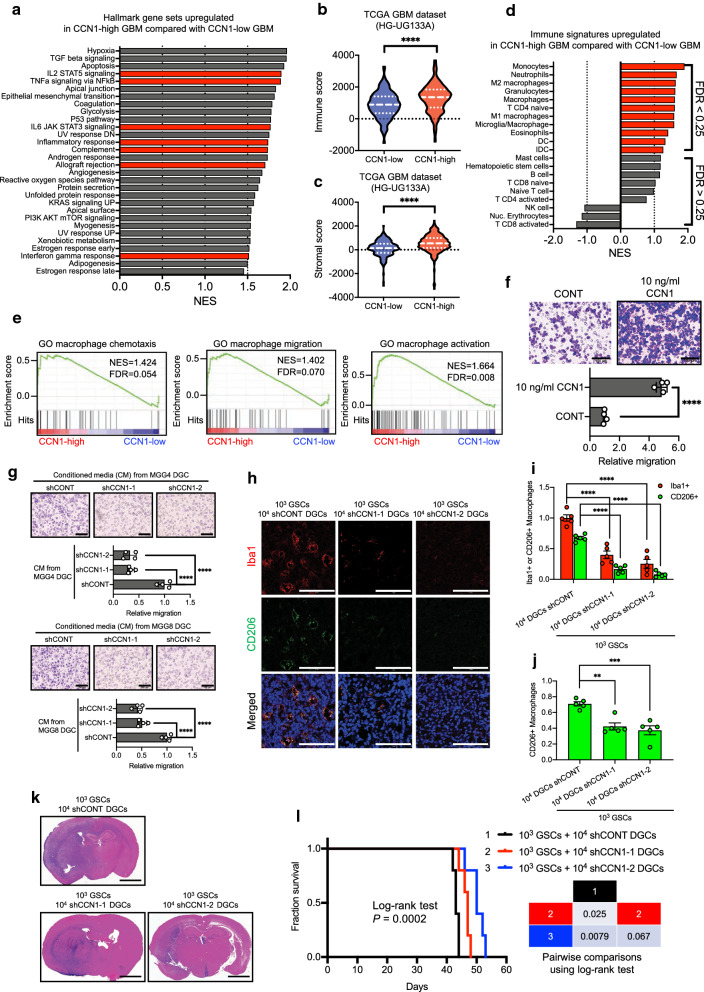
DGCs augment macrophage infiltration through secretion of CCN1 in GBM. **a** Bar graph showing the normalized enrichment score (NES) of GSEA analysis of hallmark gene sets upregulated in CCN1-high GBMs (n = 264) compared with CCN1-low GBMs (n = 264). Thirty gene sets were significantly enriched at a false discovery rate (FDR) of < 0.25 and nominal *P* value of < 0.05 in CCN1-high GBMs. TCGA GBM dataset (HG-UG133A, n = 528). Red bars indicate signatures relate to immune responses. **b** Immune score of CCN1-high (n = 264) and CCN1-low (n = 264) GBMs in TCGA GBM dataset (HG-UG133A, n = 528). Violin plots represent the median (thick dotted line) and quartiles (dotted line). ****P < 0.0001, Student’s t-test. **c** Stromal score of CCN1-high and CCN1-low GBMs in TCGA GBM dataset (HG-UG133A). Violin plots represent the median (thick dotted line) and quartiles (dotted line). ****P < 0.0001, Student’s t-test. **d** GSEA analysis of various types of immune cell signatures upregulated in CCN1-high GBMs (n = 264) compared with CCN1-low GBMs (n = 264) in TCGA GBM dataset (HG-UG133A, n = 528). Red bars indicate FDR < 0.25. **e** GSEA analysis of macrophage-related signatures upregulated in CCN1-high GBMs (n = 264) compared with CCN1-low GBMs (n = 264) in TCGA GBM dataset (HG-UG133A, n = 528). FDR < 0.25 was defined as significantly enriched. **f** Representative image (upper panel) and quantification (lower panel) of transwell analysis of U937 macrophages upon stimulation with or without recombinant CCN1 (10 ng/ml). Scale bar, 100 μm. n = 4 biological replicates, mean ± SEM, ****P < 0.0001, Student’s t-test. CONT: control. **g** Representative image (upper panel) and quantification (lower panel) of transwell analysis of U937 macrophages upon stimulation with conditioned medium (CM) from DGCs (MGG4 and MGG8 DGCs) transduced with shCONT or shCCN1. Scale bar, 100 μm. n = 4 biological replicates, mean ± SEM, ****P < 0.0001, one-way ANOVA with Tukey’s multiple comparisons test. **h** Representative confocal images of tumor-bearing brains harvested at 30 days after implantation of 1 × 10^3^ GSCs plus 1 × 10^4^ matched DGCs transduced with shCONT or 1 × 10^3^ GSCs plus 1 × 10^4^ DGCs transduced with shCCN1 derived from MGG8. Scale bar, 100 µm. Iba1 (red), CD206 (green), and DAPI (blue). **i** Quantitation of pan-macrophages (Iba1^+^) and M2 macrophages (CD206^+^) densities in xenografts formed by 1 × 10^3^ GSCs plus 1 × 10^4^ matched DGCs transduced with shCONT or 1 × 10^3^ GSCs plus 1 × 10^4^ DGCs transduced with shCCN1 derived from MGG8 cells. The total number of macrophages was counted in five randomly selected fields per sample. n = 5 biological replicates, mean ± SEM, ****P < 0.0001, one-way ANOVA with Tukey’s multiple comparisons test. **j** Quantitation of the fraction of M2 macrophages (CD206^+^). The fraction was determined by M2 macrophages (CD206^+^) among pan-macrophages (Iba1^+^) in xenografts formed by 1 × 10^3^ GSCs plus 1 × 10^4^ matched DGCs transduced with shCONT or 1 × 10^3^ GSCs plus 1 × 10^4^ DGCs transduced with shCCN1. The total number of macrophages was counted in five randomly selected fields per sample. n = 5 biological replicates. Data are represented as means ± SEM. **P < 0.01, ***P < 0.001, one-way ANOVA with Tukey’s multiple comparisons test. **k** Representative H&E stainings of tumor-bearing brains harvested at 30 days after implantation of 1 × 10^3^ GSCs plus 1 × 10^4^ matched DGCs transduced with shCONT or 1 × 10^3^ GSCs plus 1 × 10^4^ DGCs transduced with shCCN1 derived from MGG8. Scale bars, 2000 µm. **l** Kaplan–Meier (left) and log-rank *P* value (right) analyses of mice bearing orthotopic xenografts of 1 × 10^3^ GSCs plus 1 × 10^4^ matched DGCs transduced with shCONT or 1 × 10^3^ GSCs plus 1 × 10^4^ DGCs transduced with shCCN1 derived from MGG8 cells

To investigate the biological role of CCN1 in GBM, we explored transcriptomic data of TCGA GBM cohorts. CCN1-high GBMs exhibited prominent representation of immune response gene sets in GSEA, which included the interferon α/γ response, TNF-α/NF-κB signaling, inflammatory response, interleukin-2 (IL-2)/STAT5 signaling, and IL-6/STAT3 signaling (Fig. 6a). CCN1-high GBMs exhibited higher stromal and immune signatures and lower tumor purity than CCN1-low GBMs (Fig. 6b, c, Additional file 1: S6a). Analysis of immune cell signatures demonstrated that high CCN1 expression correlated to significant enrichment of macrophages (total M1 and M2), microglia, and monocytes (Fig. 6d). Gene ontology enrichment analysis (GOEA) of the subontologies of the Biological Process in TCGA GBM patients, which demonstrated that leukocyte migration and chemotaxis activity were CCN1-regulated processes (Additional file 1: Fig. S6b). Furthermore, the CCN1-high GBM group exhibited significant enrichment of macrophage-related gene sets (macrophage chemoattractant, migration, and activation) in the GSEA (Fig. 6e). Taken together, these in silico findings suggest a role of CCN1 in macrophage infiltration into GBM.

Next, to determine the CCN1 distribution and its correlation with macrophage infiltration into GBMs, frozen sections of tumor tissue from a mouse de novo GBM model were coimmunostained with CCN1 and M2 macrophage marker CD206. We found that tumor areas with more CD206-positive macrophage infiltration showed more CCN1 staining (Additional file 1: Fig. S6c). To directly validate the capacity of CCN1 to function as a macrophage chemoattractant, we conducted transwell migration assays using U937 macrophages and found that recombinant CCN1 protein increased U937 macrophage migration (Fig. 6f). To more strictly assess the effect of CCN1 on macrophage migration and tumor growth, we conducted conditional small hairpin RNA (shRNA)-mediated knockdown of CCN1 using lentiviral vectors in DGCs from patient-derived GBM cells (MGG4 and MGG8 DGCs) and U87ΔEGFR cells (Additional file 1: Fig. S6d). Conditioned medium (CM) from DGCs (MGG4 and MGG8 DGCs) and U87ΔEGFR cells transduced with shRNA that targeted CCN1 (shCCN1) decreased U937 macrophage migration relative to a non-targeting control shRNA (shCONT) in transwell migration assays (Fig. 6g, Additional file 1: S6e). Mice bearing xenografts formed by U87ΔEGFR cells transduced with shCONT exhibited more macrophage infiltration into tumors and shorter survival compared with those transduced with shCCN1 (Additional file 1: Fig. S6f–i). Furthermore, to address the significance of CCN1 secreted from DGCs in the mouse xenograft model, we coimplanted GSCs and DGCs transduced with shCONT or shCCN1 into the brains of immunocompromised mice. The combination of GSCs and DGCs transduced with shCCN1 led to reductions in macrophage infiltration into tumors and the tumor size compared with the combination of GSCs and DGCs transduced with shCONT (Fig. 6h–k). Mice bearing xenografts formed by GSCs in combination with DGCs transduced with shCONT had shorter survival than those bearing xenografts formed by the combination of GSCs and DGCs transduced with shCCN1 (Fig. 6l). Collectively, these results suggest that DGCs augment macrophage infiltration into tumors and tumor progression, at least in part, through secretion of CCN1.

### CCN1 secreted from DGCs augments macrophage infiltration through integrins

**Fig. 7:**
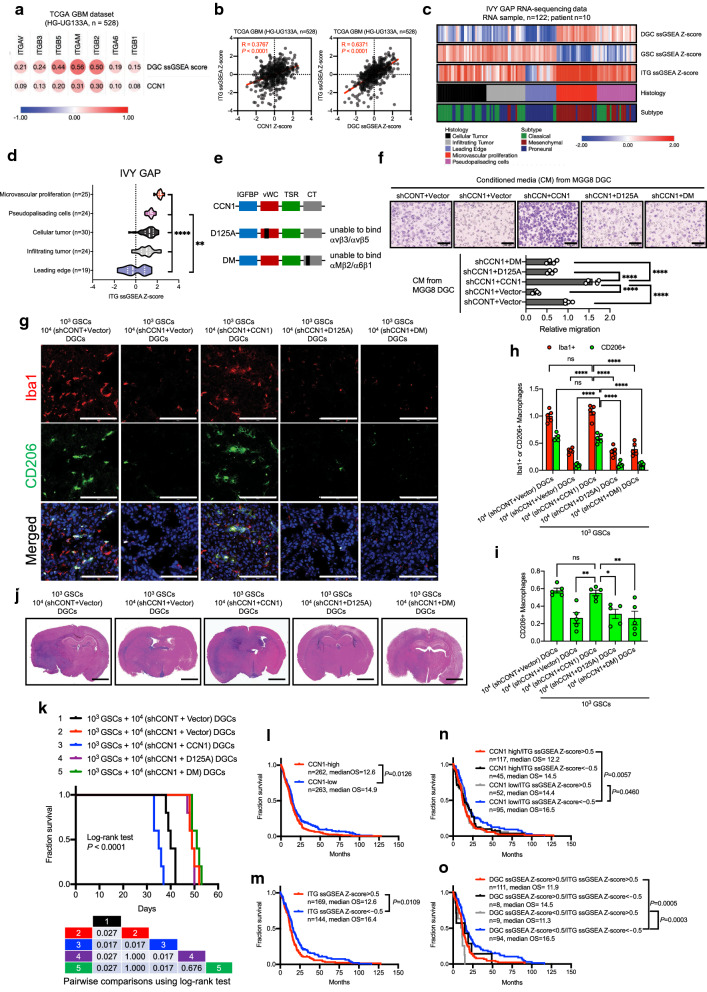
CCN1 secreted from DGCs augments macrophage infiltration through integrins. **a** Correlation analysis of mRNA expression of CCN1-binding integrins (ITGAV, ITGB3, ITGB5, ITGAM, ITGB2, ITGA6, and ITGB1) with DGC ssGSEA scores and mRNA expression of CCN1 in TCGA GBM (HG-U133A) dataset. Size and color indicate the correlation R-value. Pearson’s correlation test. **b** Correlation analysis of ITG (CCN1-binding integrin) ssGSEA scores with mRNA expression of CCN1 (left panel) and DGC ssGSEA scores (right panel) in TCGA GBM (HG-U133A) dataset. Red numbers indicate the correlation R-value and *P* value. Pearson’s correlation test. **c** Heat map shows expression of DGC, GSC, and ITG ssGSEA scores (top, second, and third from top) for each RNA sample in the anatomical structure study dataset (122 RNA sample data from 10 patients) from the IVY GAP database (columns). The corresponding GBM subtype and histology for each sample are labeled (bottom and second from bottom). **d** ssGSEA scores of ITG (CCN1-binding integrin) signature genes in multiple regions of the IVY GAP dataset (122 RNA sample data from 10 patients). Violin plots represents the median (thick dotted line) and quartiles (dotted line). **P < 0.01, ****P < 0.0001, one-way ANOVA with Tukey’s multiple comparisons test. **e** Schematic diagram showing the domain structure of wildtype CCN1 and D125A and DM mutants disrupted in binding sites for αvβ3/αvβ5 and αMβ2/α6β1 integrins, respectively. **f** Representative image (left panel) and quantification (right panel) of transwell analysis of U937 macrophages upon stimulation with conditioned medium (CM) from MGG8 DGCs with the indicated modification. Scale bar, 100 μm. n = 4 biological replicates, mean ± SEM, ****P < 0.0001, one-way ANOVA with Tukey’s multiple comparisons test. **g** Representative confocal images of tumor-bearing brains harvested at 30 days after implantation of 1 × 10^3^ GSCs plus 1 × 10^4^ matched DGCs from MGG8 cells with the indicated modification by a lentivirus. Scale bar, 100 µm. Iba1 (red), CD206 (green), and DAPI (blue). **h** Quantitation of pan-macrophages (Iba1^+^) and M2 macrophages (CD206^+^) densities in xenografts formed by 1 × 10^3^ GSCs plus 1 × 10^4^ matched DGCs from MGG8 cells with the indicated modification by a lentivirus. The total number of macrophages was counted in five randomly selected fields per sample. n = 5, mean ± SEM, ****P < 0.0001, ns: not significant, one-way ANOVA with Tukey’s multiple comparisons test. **i** Quantitation of the fraction of M2 macrophages (CD206^+^). The fraction was determined by M2 macrophages (CD206^+^) among pan-macrophages (Iba1^+^) in xenografts formed by 1 × 10^2^ GSCs plus 1 × 10^4^ matched DGCs from MGG8 cells with the indicated modification by a lentivirus. The total number of macrophages was counted in five randomly selected fields per sample. n = 5 biological replicates. Data are represented as means ± SEM. *P < 0.05, **P < 0.01, ns: not significant, one-way ANOVA with Tukey’s multiple comparisons test. **j** Representative H&E stainings of tumor-bearing brains harvested at 30 days after implantation of 1 × 10^3^ GSCs plus 1 × 10^4^ matched DGCs from MGG8 cells with the indicated modification by a lentivirus. Scale bars, 2000 µm. **k** Kaplan–Meier (upper) and log-rank *P* value (bottom) analyses of mice bearing orthotopic xenografts of 1 × 10^3^ GSCs plus 1 × 10^4^ matched DGCs from MGG8 cells with the indicated modification by a lentivirus. **l** Kaplan–Meier analyses between patients in CCN1-high and CCN1-low groups on the basis of median mRNA expression in TCGA GBM dataset (HG-UG133A). Log-rank *P* value analyses. **m** Kaplan–Meier analyses of patients in TCGA GBM dataset (HG-UG133A) on the basis of ssGSEA scores of ITG (CCN1-binding integrin) signature genes. Log-rank *P* value analyses. **n** Negative correlation between expression of CCN1 combined with ITG (CCN1-binding integrin) signature genes and overall patient survival in TCGA GBM (HG-U133A) dataset. Log-rank *P* value analyses. **o** Negative correlation between expression of DGC-signature genes combined with ITG (CCN1-binding integrin) signature genes and overall patient survival in TCGA GBM (HG-U133A) dataset. Log-rank *P* value analyses

CCN1 functions through binding to at least five different integrins (αvβ3, αvβ5, α6β1, αIIbβ3, and αMβ2) [31]. The expression of these CCN1-binding integrins was positively correlated to DGC signature genes and CCN1 in TCGA GBM (HG-U133A) dataset (Fig. 7a, Additional file 1: S7a). Furthermore, we calculated ssGSEA scores of the ITG signature (expression of CCN1-binding integrins) for individual GBM samples. The ITG signature genes were also positively correlated to DGC signature genes and CCN1 (Fig. 7b). In the IVY GAP dataset, ITG signature genes were particularly enriched in regions of microvascular proliferation, while expression of ITG signature genes was lower in leading edge regions (Fig. 7c, d). Next, we investigated deposited single cell RNA-sequencing data from GBM tumors and confirmed that CCN1 was expressed in neoplastic cells and CCN1-binding integrins were expressed in myeloid cells (Additional file 1: Fig. S7b).

To investigate whether DGC-derived CCN1-mediated macrophage infiltration into GBM may be mediated by integrins, we cloned lentiviral vectors harboring wildtype (WT) CCN1 or mutant CCN1 (D125A [14], a CCN1 mutant defective for binding αvβ3/αvβ5 integrins, and DM [32], a CCN1 mutant defective for binding αMβ2/α6β1 integrins) (Fig. 7e). We found that rescue of endogenous CCN1, which was knocked down by shRNA that targeted 3ʹ-untranslated regions (UTRs), by exogenously transduced lentiviruses that harbored WT CCN1 restored macrophage infiltration in vitro and in vivo, increased the tumor size, and reduced animal survival, but not the empty vector control or CCN1 mutants defective for integrin binding (Fig. 7g–k, Additional file 1: S7c–g).

To determine the clinical relevance of the DGC signature and CCN1-binding integrins in GBM patients, we re-examined TCGA GBM (HG-U133A) dataset. High expression of CCN1 and the ITG signature (expression of CCN1-binding integrins) was associated with a poor prognosis (Fig. 7l, m). Furthermore, expression of the DGC signature combined with CCN1 or the ITG-signature were negatively correlated to overall patient survival (Fig. 7n, o).

Taken together, our data elucidated the biological roles of DGCs, especially in the tumor microenvironment. DGCs augment macrophage infiltration through CCN1 to promote tumor progression of GBM.

## Discussion

In this study, we extracted DGC signature genes from transcriptomic profiles of matched pairs of in vitro GSCs and DGCs models. We evaluated the DGC signature genes in single cell RNA-sequencing data, which confirming the presence of cell subpopulations emulated by in vitro culture models within a primary tumor. We found that the DGC gene signature was correlated to macrophage-related genes, the mesenchymal subtype signature, and poor survival. DGCs exhibited significant enrichment of YAP/TAZ/TEAD activity compared with GSCs. We elucidated that DGCs promote macrophage recruitment into GBM tissue through secretion of CCN1.

The significance of GSCs is undeniable considering their capacity for self-renewal, differentiation, and tumor propagation in vivo, and their contribution to therapeutic resistance, immune escape, and angiogenesis [1, 23]. The focus on GSCs has overlooked the importance of differentiated progeny such as DGCs. A recent study has shown that DGCs also contribute to tumor progression in concert with GSCs through a paracrine loop [57]. However, the roles of DGCs in the tumor microenvironment of GBM remain obscure. In the present study, we focused on the significant correlation between DGC and macrophage signature genes, and established that DGCs secrete CCN1 to promote macrophage infiltration into the GBM tumor microenvironment. In addition to CCN1, CLCF1, a secreted protein included among the DGC signature genes, is implicated in macrophage activation [37]. Furthermore, a recent study of medulloblastoma models has also shown that tumor-derived astrocytes, differentiated progeny from tumor progenitors, induce polarization of resident brain microglia towards protumorigenic macrophages by secreting IL-4 [59]. These findings suggest that not only tumor progenitors such as GSCs, but also differentiated progeny such as DGCs play an essential role in shaping the complex tumor immune microenvironment by promoting macrophage infiltration.

The mesenchymal signature of GBM can be shaped by several factors such as stromal cells, accumulated mutations in tumor cells, the cell of origin, anatomical location/tumor microenvironments, and therapy-induced mesenchymal transition [4]. The presence of macrophages/microglia is associated with the mesenchymal subtype of GBM [6, 34, 56]. Genetic deficiency of NF1 attracts macrophages/microglia into tumors and a macrophage/microglia-rich microenvironment also induces a mesenchymal tumor cell phenotype [56]. We found that DGCs themselves had a mesenchymal gene signature and the DGC signature was anatomically enriched in perinecrotic/hypoxic (pseudopalisading) regions and microvascular proliferative regions. These findings are consistent with prior reports indicating that the mesenchymal signature is enriched in perinecrotic/hypoxic regions and microvascular proliferative regions [41]. Thus, DGCs may induce a mesenchymal phenotype by attracting macrophages.

DGCs do not form tumors when implanted alone [51, 57]. This suggest that retrograde dedifferentiation from DGCs to GSCs essentially cannot occur in a tumor [51]. DGCs successfully implant in brains of recipient mice only when cotransplanted with GSCs, which contribute to GSC-dependent tumor progression [57]. We also obtained the same result in the present study. Considering our results that DGCs had a mesenchymal gene signature and GSCs had a proneural gene signature when compared each other, increased intratumoral heterogeneity at the earliest time of tumor initiation by cotransplantation of DGCs and GSCs may accelerate tumor progression. This may be consistent with the result of a previous study in which proneural GBM patients with a high proportion of alternate subtype tumor cells had significantly worse outcomes compared with the pure proneural subtype [38]. Combination therapies that simultaneously target both DGCs and GSCs may be needed to overcome this intratumoral heterogeneity of GBMs.

In GBMs, TAZ is a transcriptional coactivator that drives the gene expression program of mesenchymal differentiation in a TEAD-dependent fashion [5]. We found that DGCs exhibited significant enrichment of YAP/TAZ/TEAD activity compared with GSCs. Previous studies have reported that serum stimulation in vitro activates AP-1, a transcriptional partner of TEADs, and YAP/TAZ [61, 62, 64]. Additionally, YAP/TAZ are implicated in mechanotransduction. When cells are cultured on a stiff substrate, YAP/TAZ localize in the nucleus and become transcriptionally active [17]. Our results are reasonable considering the cell culture conditions under which GSCs grew as spheres in serum-free conditions and DGCs expanded as adherent monolayers under serum-containing conditions. Elevated tissue tension induces a mesenchymal-like phenotype in GBM [3]. Thus, elevated stiffness may promote YAP/TAZ/TEAD activation of DGCs in GBM tissue.

CCN1, a transcriptional target of YAP/TAZ/TEAD, is secreted from various cell type, including, tumor cells, endothelial cells, fibroblasts, and smooth muscle cells [19, 36, 40]. CCN1 has been implicated in various cellular processes including leukocyte infiltration, inflammatory process, angiogenesis, and adhesion [19, 25, 30, 36, 53]. Moreover, we and others have revealed an elevation of CCN1 expression and its correlation with a poor prognosis in various tumors including GBM [9, 27, 35, 36]. However, which subpopulations of heterogeneous GBM cells secrete CCN1 and its detailed role in the heterogeneous GBM microenvironment has not been fully elucidated. We found that CCN1 was secreted abundantly from a population of DGCs, but not GSCs, which played critical roles in shaping the mesenchymal phenotype through macrophage infiltration into GBM tissue.

The gene signature of in vitro culture models should be interpreted with caution because in vitro DGC and GSC models do not fully recapitulate the heterogeneity of tumor cells within primary GBMs. However, a prior study applied a stemness signature of in vitro culture models to GBM single cell transcriptional profiles and revealed the existence of cell subpopulations emulated by in vitro culture models within a primary tumor [38]. Interestingly, each in vitro model represents phenotypic extremes of the stemness gradients within GBM tumors [38]. Using other deposited GBM single cell RNA-sequencing data, we also found clear gradients of DGC and GSC signatures and negative correlations between these signatures in each sample. These results reflect the validity of our approach using the DGC gene signature of in vitro culture models to investigate the biological roles of DGCs in GBMs.

Stem cell-like tumor-initiating cells expressing markers of proneural and mesenchymal transcriptomic subtypes (proneural and mesenchymal GSCs, respectively) have been reported in GBM [29]. In the present study, we used the proneural subtype of GSC models [51]. The biological phenotype and anatomical distribution of proneural and mesenchymal GSCs are different [29]. Accordingly, in the future, studies that focus on the differences among proneural GSCs, mesenchymal GSCs, and DGCs from each subtype of GSCs are needed.

A limitation of the present study is that immune responses, including microglia/macrophages, are different in immunocompetent and immunodeficient mice. BALB/c-nu/nu mice have impaired T-cell functions and an active macrophage system [12]. Therefore, in the future, we should perform in vivo experiments using immunocompetent syngeneic mouse models.

In conclusion, our results reveal that DGCs contribute GSC-dependent tumor progression by shaping a mesenchymal microenvironment via macrophage infiltration and provide a new insight into the complex GBM microenvironment consisting of heterogeneous mixtures of cells.

## Supplementary Information

**Additional file 1**. Supplementary figures and figure legends. Fig. S1–S7.
