# Supplementary material for: Differentiated glioblastoma cells accelerate tumor progression by shaping the tumor microenvironment via CCN1-mediated macrophage infiltration
Source: Acta Neuropathol Commun. 2021 Feb 22;9:29. doi: 10.1186/s40478-021-01124-7 (PMC7898455; doi:10.1186/s40478-021-01124-7)
Supplement: Supplementary file 1 — Additional file 1. Supplementary figures and figure legends. Fig. S1–S7. [file 40478_2021_1124_MOESM1_ESM.pdf]

SUPPLEMENTARY MATERIAL

Differentiated glioblastoma cells accelerate tumor progression by shaping the tumor microenvironment via CCN1-mediated macrophage infiltration

Atsuhito Uneda[1,2]; Kazuhiko Kurozumi[1,3,#]; Atsushi Fujimura[2,4,#]; Kentaro Fujii[1]; Joji Ishida[1]; Yosuke Shimazu[1]; Yoshihiro Otani[1]; Yusuke Tomita[1]; Yasuhiko Hattori[1]; Yuji Matsumoto[1]; Nobushige Tsuboi[1]; Keigo Makino[1]; Shuichiro Hirano[1]; Atsunori Kamiya[2]; Isao Date[1]

[1] Department of Neurological Surgery, Okayama University Graduate School of Medicine, Dentistry, and Pharmaceutical Sciences, 2-5-1 Shikata-cho, Kita-ku, Okayama 700-8558, Japan

[2] Department of Cellular Physiology, Okayama University Graduate School of Medicine, Dentistry and Pharmaceutical Sciences, 2-5-1 Shikata-cho, Kita-ku, Okayama 700-8558, Japan

[3] Department of Neurosurgery, Hamamatsu University School of Medicine, 1-20-1 Handayama, Higashi-ku, Hamamatsu-city, Shizuoka 431-3139, Japan.

[4] Neutron Therapy Research Center, Okayama University, 2-5-1 Shikata-cho, Kita-ku, Okayama 700-8558, Japan

[#]These authors jointly supervised this study

Corresponding authors:

Kazuhiko Kurozumi, M.D., Ph.D.

E-mail: kurozu20@hama-med.ac.jp

Tel: +81-53-435-2283

Fax: +81-53-435-2282

Atsushi Fujimura, M.D., Ph.D.

E-mail: atsushi.fujimura@okayama-u.ac.jp

Tel: +81-86-235-7105

Fax: +81-86-235-7111

Supplementary figure legends

Fig. S1 (Related to Fig. 1)

a

Top 50 DGC signature genes

COL5A2	MYH9	NPC2	PXK	LRP10
FAP	EMP1	MYOF	STC2	PARVA
LTBP2	IGFBP7	LOXL2	CYR61	VEGFC
AHNAK	HEBP2	POLR2B	GALC	ARHGAP24
CLCF1	ANXA2P3	COL5A1	BICC1	MYD88
GPR176	PHF11	TNFSF4	PGRMC2	BEND6
ANXA2P2	RRAS	HLX	CD109	CD24
ANXA2	FAM180A	AHNAK2	OBFC2A	LY96
CASP4	CAV2	CSRP1	PALLD	C10orf26
COL6A1	ANXA2P1	SPARC	LOC100132891	CALCOCO2

b

Top 50 GSC signature genes

ABAT	TMPRSS5	BK250D10.8	MAST1	PREX1
SAPCD2	NOTCH1	LPHN3	RLBP1	MMP15
PTPRZ1	DHODH	NDRG2	TRAF4	SCRG1
HNRNPA3	MAGED2	TPD52	CAMKV	NAT8L
ZNF276	SCARB1	FREM2	SBK1	MYH14
LOC100499467	ISYNA1	SOX5	PEG3	TUBB2B
SPTBN2	HES6	IQCK	GNG4	GLB1L2
MYBL2	LOC729080	RNF165	LAMC3	S100B
PDE3B	CADM4	NCAN	GLDC	AIF1L
GNG7	ARHGDIG	TEX15	FAM198A	COL9A3

Fig. S1 (Related to Fig. 1)

a) List of the top 50 genes upregulated in DGCs.

b) List of the top 50 genes upregulated in GSCs.

Fig. S2 (Related to Fig. 2)

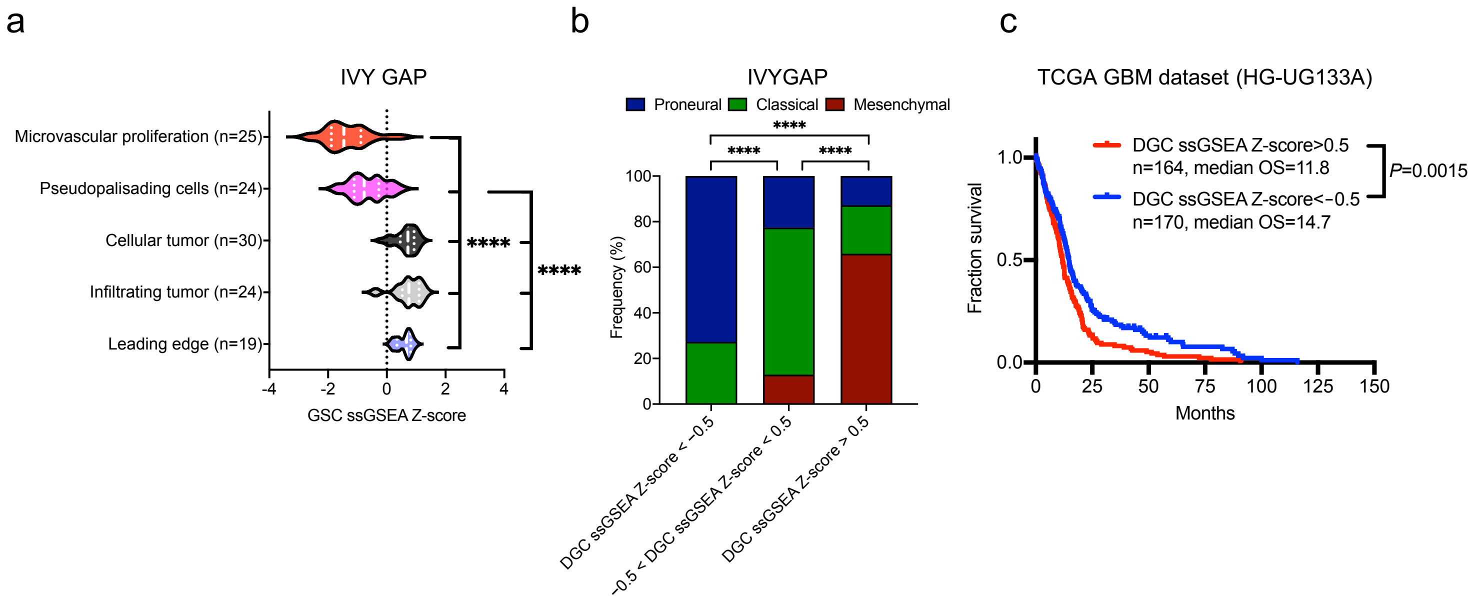

Fig. S2 (Related to Fig. 2)

a) ssGSEA scores of GSC signature genes in multiple regions of IVY GAP data (122 RNA sample data from 10 patients). Violin plots represent the median (thick dotted line) and quartiles (dotted line). ****$P < 0.0001$, one-way ANOVA with Tukey's multiple comparisons test.

b) Molecular subtype distribution among three groups on the basis of ssGSEA scores of the DGC signature in IVY GAP data (122 RNA sample data from 10 patients). ****$P < 0.0001$, chi-squared test.

c) Kaplan–Meier analyses of patients in TCGA GBM dataset (HG-UG133A) on the basis of ssGSEA scores of DGC signature genes. Log-rank *P*-value analyses.

Fig. S3 (Related to Fig. 3)

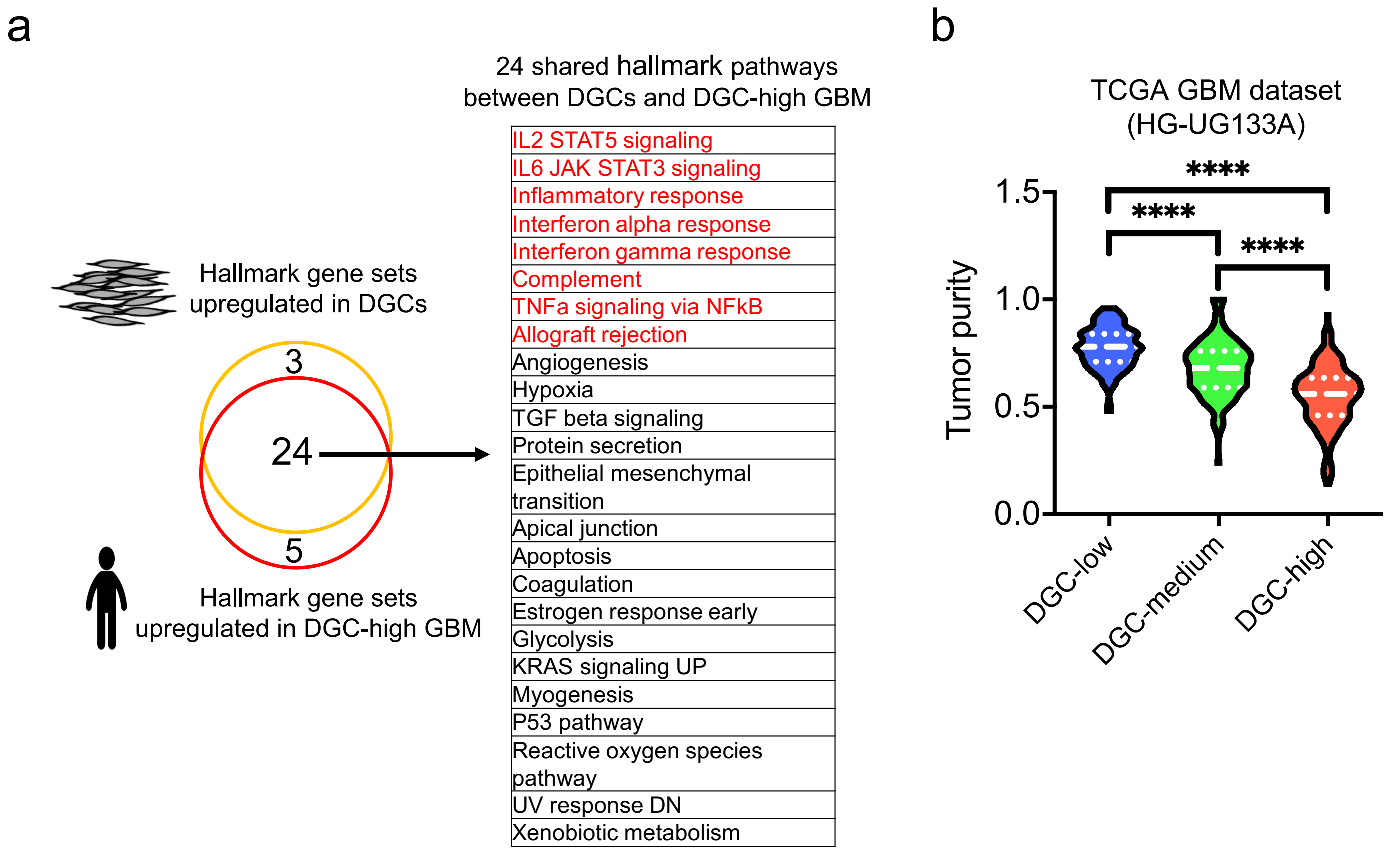

Fig. S3 (Related to Fig. 3)

a) Venn diagram showing the shared hallmark pathways of DGCs and DGC-high GBM in TCGA. The table shows the 24 shared hallmark pathways of DGCs and DGC-high GBM. Red indicates signatures related to immune responses.

b) Tumor purity score of DGC-high, -medium, and -low patients in TCGA GBM dataset (HG-UG133A). Violin plots represent the median (thick dotted line) and quartiles (dotted line). ****P < 0.0001, one-way ANOVA with Tukey's multiple comparisons test.

Fig. S4 (Related to Fig. 4)

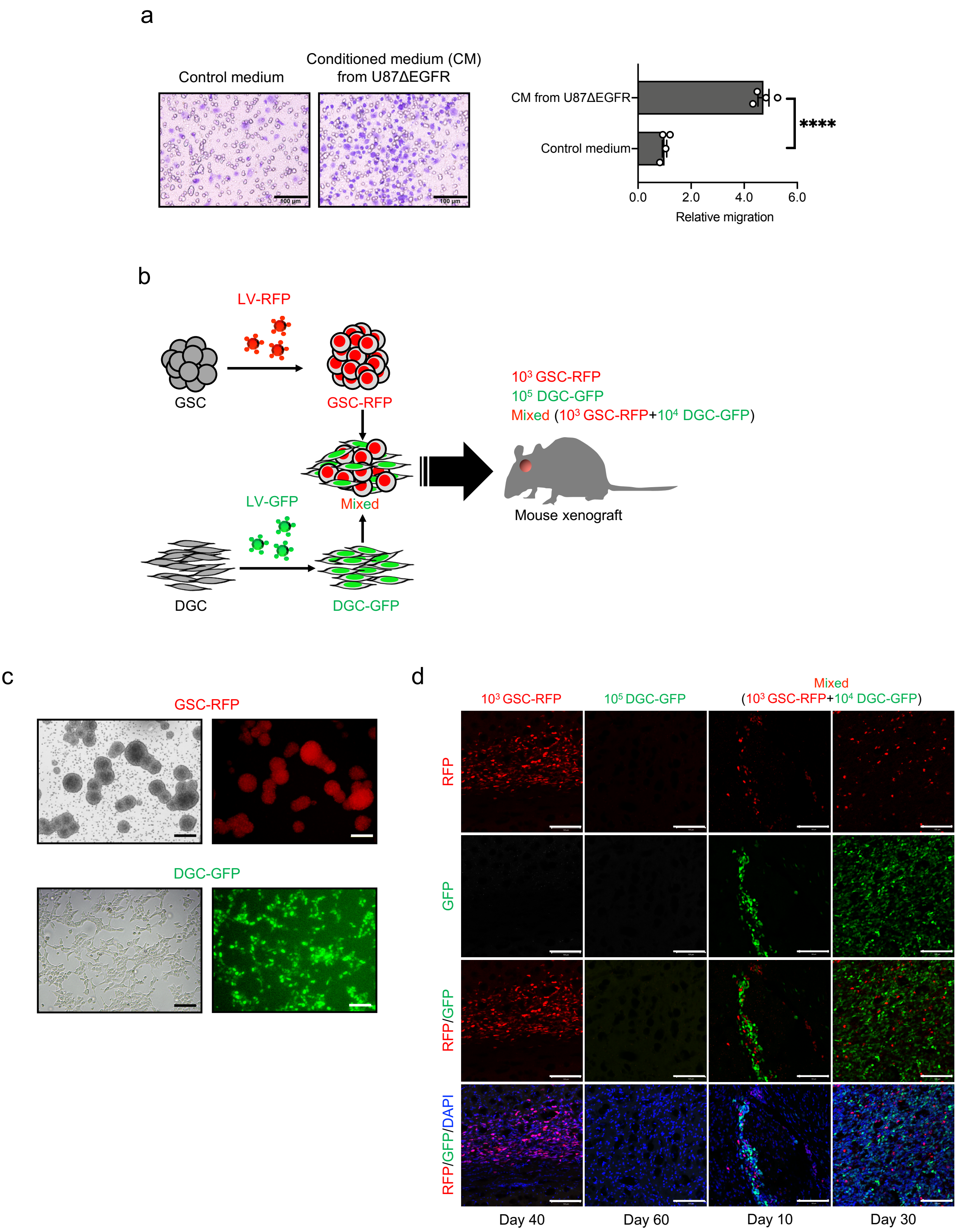

Fig. S4 (Related to Fig. 4)

a) Representative image (left panel) and quantification (right panel) of transwell analysis of U937 macrophages upon stimulation with control medium or conditioned medium (CM) from U87ΔEGFR cells. Scale bar, 100 μm. n = 4 biological replicates, mean ± SEM, ***P < 0.001, Student's t-test.

b) Schematic illustration of experiments performed with retroviral tracing of tumor cells. Tumor cells were labeled with fluorescent proteins (GFP or RFP) by lentiviral vectors (LVs). GSCs labeled with RFP alone, DGCs labeled with GFP alone, or their combination were injected into the brains of recipient mice.

c) Representative images of GSCs labeled with RFP and DGCs labeled with GFP derived from MGG8 cells. Scale bar, 100 μm.

d) Representative confocal images of tumor-bearing brains harvested after implantation of 1×10^3 GSC-RFP alone, 1×10^5 DGC-GFP alone, or both (1×10^3 GSC-RFP plus 1×10^4 matched DGC-GFP cells) derived from MGG8 cells. Scale bar, 100 μm. The time between implantation and tissue harvesting is shown below. RFP (red), GFP (green), and DAPI (blue).

Fig. S5 (Related to Fig. 5)

a

Homer *de novo* Motif Enrichment Results
(DGCspecific)

Rank	Best Match	*P*-value
1	Fra1(bZIP)	1E-315
2	TEAD4	1E-108
3	RUNX1	1E-54
4	MF0010.1_Homeobox_class	1E-43
5	Etv2(ETS)	1E-36

Total target sequences = 5623
Total background sequences = 44227

Homer Known Motif Enrichment Results
(DGCspecific)

Rank	Motif Name	*P*-value
1	BATF(bZIP)	1E-313
2	AP-1(bZIP)	1E-312
3	Fra1(bZIP)	1E-309
4	Atf3(bZIP)	1E-306
5	JunB(bZIP)	1E-303
6	Fra2(bZIP)	1E-291
7	Fosl2(bZIP)	1E-252
8	Jun-AP1(bZIP)	1E-204
9	Bach2(bZIP)	1E-111
10	TEAD1(TEAD)	1E-95
11	TEAD3(TEA)	1E-90
12	TEAD4(TEA)	1E-77
13	TEAD(TEA)	1E-76
14	NF-E2(bZIP)	1E-59
15	Nrf2(bZIP)	1E-59
16	TEAD2(TEA)	1E-56
17	Bach1(bZIP)	1E-53
18	RUNX1(Runt)	1E-52
19	MafK(bZIP)	1E-48

Total Target Sequences = 5625
Total Background Sequences = 44283

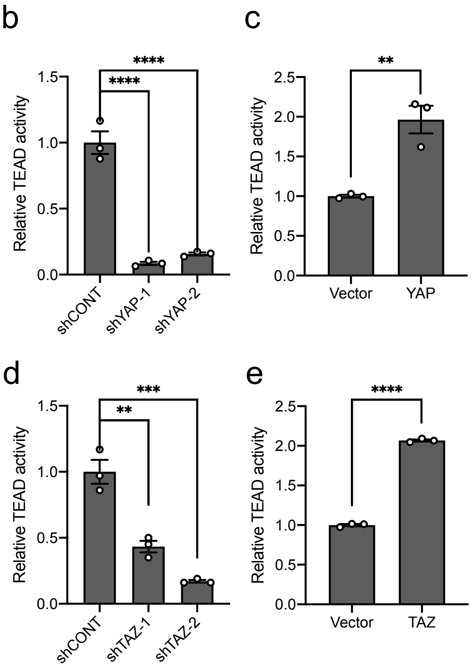

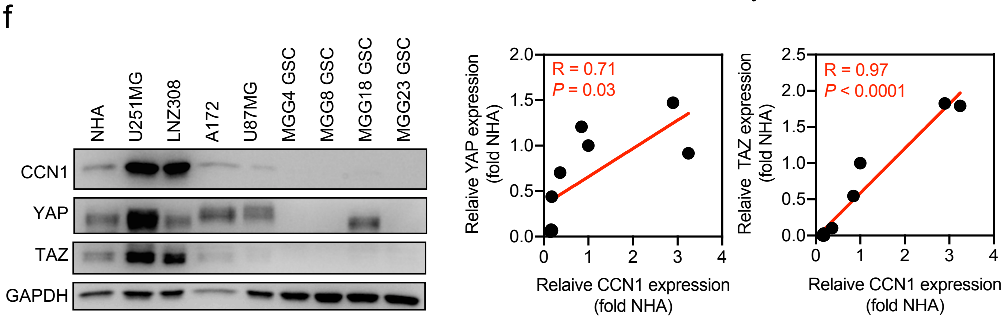

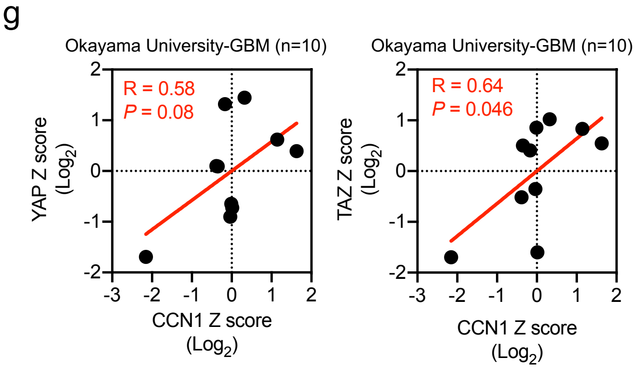

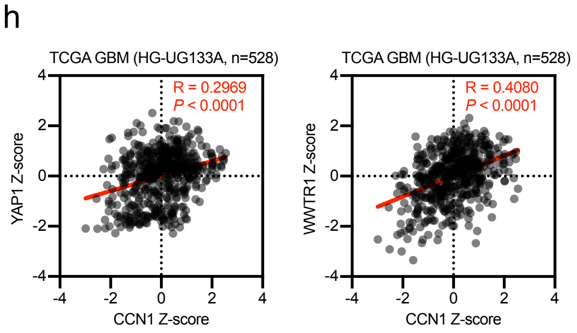

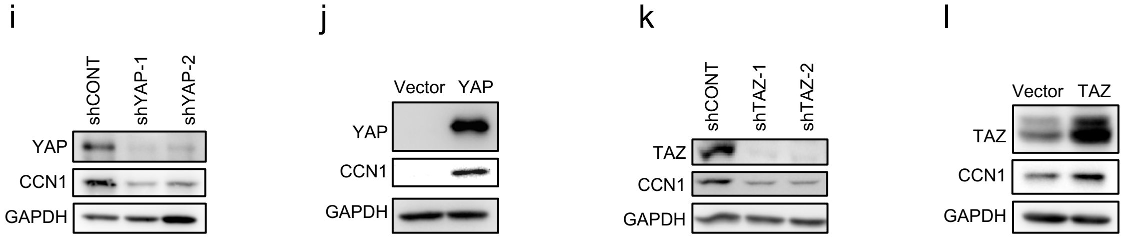

Fig. S5 (Related to Fig. 5)

a) Tables showing the ranking of *de novo* and known motifs of DGC-specific enhancers defined in Figure 5a,b. Red indicates the TEAD transcription factor family (TEAD1–4) and activator protein-1 (AP-1, dimer of JUN and FOS proteins).

b) Quantification of the TEAD luciferase reporter assay of U251MG cells transduced with lentiviral vectors expressing a non-targeting control shRNA (shCONT) or one of two shRNAs that targeted YAP (shYAP). Data are presented as the mean ± SEM of three independent experiments. ****$P < 0.0001$.

c) Quantification of the TEAD luciferase reporter assay of U87MG cells transduced with an empty lentiviral vector or lentiviral vector expressing YAP. Data are presented as the mean ± SEM of three independent experiments. **$P < 0.01$.

d) Quantification of the TEAD luciferase reporter assay of U251MG cells transduced with lentiviral vectors expressing a non-targeting control shRNA (shCONT) or one of two shRNAs that targeted TAZ (shTAZ). Data are presented as the mean ± SEM of three independent experiments. **$P < 0.01$, ***$P < 0.001$.

e) Quantification of the TEAD luciferase reporter assay of U87MG cells transduced with an empty lentiviral vector or a lentiviral vector expressing TAZ. Data are presented as the mean ± SEM of three independent experiments. ****$P < 0.0001$.

f) Immunoblot (left) and correlations (right) of CCN1, YAP, and TAZ in cell lysates of NHAs (normal human astrocytes), U251MG, LNZ308, A172, and U87MG cells, and GSCs of patient-derived GBM cell lines (MGG4, MGG8, MGG18, and MGG23). GAPDH was used as a loading control. Red numbers indicate the correlation R-value. Pearson's correlation test.

g) Correlation between mRNA expression of CCN1, YAP1 (gene name of YAP), and WWTR1 (gene name of TAZ) in GBM tumors from Okayama University (n = 10) assessed by qRT-PCR. Red numbers indicate the correlation R-value. Pearson's correlation test.

h) Correlation between CCN1, YAP1, and WWTR1 (gene name of TAZ) in the TCGA GBM (HG-U133A) dataset. Red numbers indicate the correlation R-value. Pearson's correlation test.

i) Representative immunoblot of YAP and CCN1 in lysates of U251MG cells transduced with lentiviral vectors expressing a non-targeting control shRNA (shCONT) or one of two shRNAs that targeted YAP (shYAP). GAPDH was used as a loading control.

j) Representative immunoblot of YAP and CCN1 in lysates of U87MG cells transduced with a control lentiviral vector or a lentiviral vector expressing YAP. GAPDH was used as a loading control.

k) Representative immunoblot of TAZ and CCN1 in lysates of U251MG cells transduced with lentiviral vectors expressing a non-targeting control shRNA (shCONT) or one of two shRNAs that targeted TAZ (shTAZ). GAPDH was used as a loading control.

l) Representative immunoblot of TAZ and CCN1 in lysates of U87MG cells transduced with a control lentiviral vector or a lentiviral vector expressing TAZ. GAPDH was used as a loading control.

Fig. S6 (Related to Fig. 6)

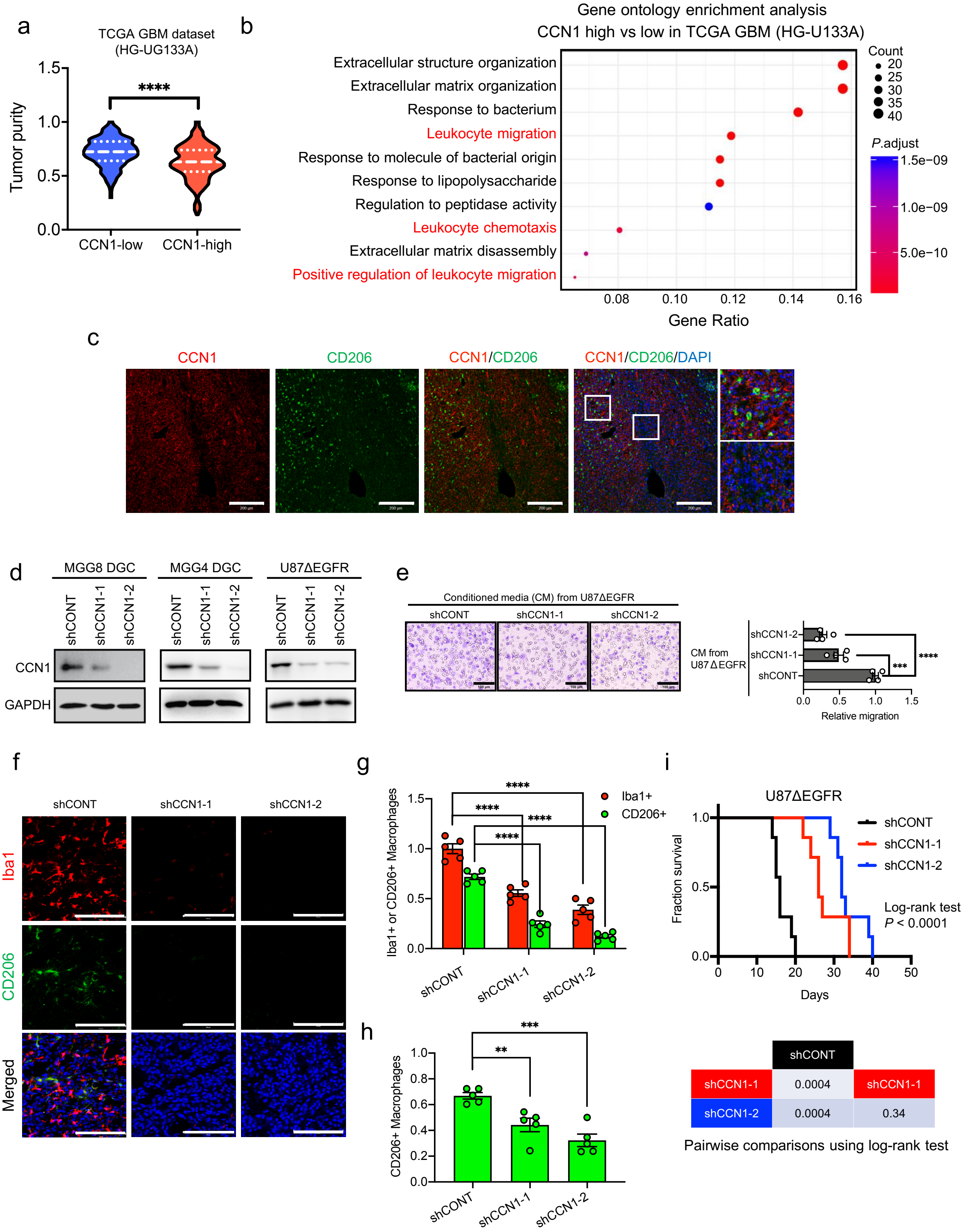

	shCONT	
shCCN1-1	0.0004	shCCN1-1
shCCN1-2	0.0004	0.34

Pairwise comparisons using log-rank test

Fig. S6 (Related to Fig. 6)

a) Tumor purity score of CCN1-high (n = 264) and CCN1-low (n = 264) GBMs in TCGA GBM dataset (HG-UG133A, n = 528). Violin plots represent the median (thick dotted line) and quartiles (dotted line). ****$P < 0.0001$, one-way ANOVA with Tukey's multiple comparisons test.

b) Gene Ontology enrichment analysis of the role of CCN1 in leukocyte/myeloid migration. Gene Ontology enrichment analysis of the function (Biological process sub-ontology) of CCN1 in TCGA GBM patients (HG-UG133A, n = 528). The top 10 enriched pathways are shown and the function related to leukocyte/myeloid cell migration and chemotaxis is indicated.

c) Representative confocal images of tumor tissue from the mouse de novo GBM model in which lentiviruses harboring H-Ras and shP53 were stereotactically injected into the hippocampus of GFAP-cre mice. Scale bar, 200 µm. Iba1 (red), CD206 (green), and DAPI (blue). Far right panels are high magnifications of the rectangle area (upper: CCN1-high area; lower: CCN1-low area).

d) Protein levels of CCN1 were assessed by immunoblotting in DGCs (MGG4 and MGG8 DGCs) and U87ΔEGFR transduced with shCONT or shCCN1. GAPDH was used as a loading control.

e) Representative image (upper panel) and quantification (lower panel) of transwell analysis of U937 macrophages upon stimulation with conditioned medium (CM) from U87ΔEGFR cells transduced with shCONT or shCCN1. Scale bar, 100 µm. n = 4 biological replicates, mean ± SEM, ****$P < 0.0001$, one-way ANOVA with Tukey's multiple comparisons test.

f) Representative confocal images of tumor-bearing brains harvested at 10 days after implantation of U87ΔEGFR cells transduced with shCONT (control) or shCCN1. Scale bar, 100 µm. Iba1 (red), CD206 (green), and DAPI (blue).

g) Quantitation of pan-macrophages ($Iba1^+$) and M2 macrophages ($CD206^+$) densities in xenografts formed by U87ΔEGFR cells transduced with shCONT or shCCN1. n = 5 biological replicates, mean ± SEM, ****$P < 0.0001$, one-way ANOVA with Tukey's multiple comparisons test.

h) Quantitation of the fraction of M2 macrophages ($CD206^+$). The fraction was determined by M2 macrophages ($CD206^+$) among pan-macrophages ($Iba1^+$) in xenografts derived from U87ΔEGFR cells transduced with shCONT or shCCN1. Data are represented as means ± SEM. **$P < 0.01$, ***$P < 0.001$, one-way ANOVA with Tukey's multiple comparisons test.

i) Kaplan–Meier (upper) and log-rank *P*-value (lower) analyses of mice bearing orthotopic xenografts of U87ΔEGFR cells transduced with shCONT or shCCN1.

Fig. S7 (Related to Fig. 7)

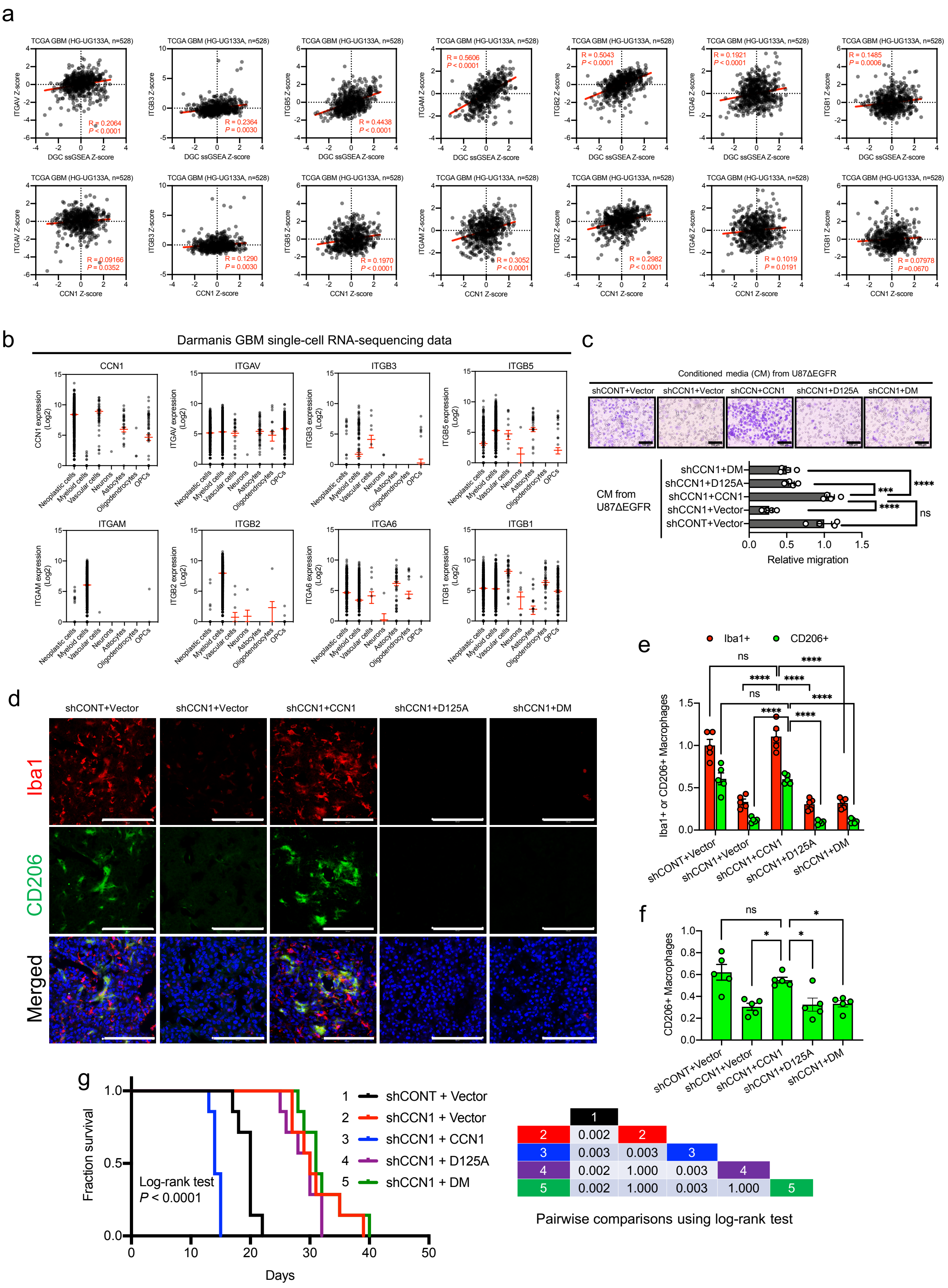

Fig. S7 (Related to Fig. 7)

a) Correlation analysis of mRNA expression of CCN1-binding integrins (ITGAV, ITGB3, ITGB5, ITGAM, ITGB2, ITGA6, and ITGB1) with DGC ssGSEA scores and mRNA expression of CCN1 in TCGA GBM (HG-U133A) dataset. Red numbers indicate the correlation R-value and *P*-value. Pearson's correlation test.

b) Dot plot showing mRNA expression of CCN1 and CCN1-binding integrins (ITGAV, ITGB3, ITGB5, ITGAM, ITGB2, ITGA6, and ITGB1) in various type of individual cells in single cell RNA-sequencing data. Data are presented as the mean ± SEM. NCBI Gene Expression Omnibus GSE84465.

c) Representative image (upper panel) and quantification (lower panel) of transwell analysis of U937 macrophages upon stimulation with conditioned medium (CM) from U87ΔEGFR cells with the indicated modification. Scale bar, 100 μm. n = 4 biological replicates, mean ± SEM, $^{***}P < 0.001$, $^{****}P < 0.0001$, ns: not significant, one-way ANOVA with Tukey's multiple comparisons test.

d) Representative confocal images of tumor-bearing brains harvested at 10 days after implantation of U87ΔEGFR with the indicated modification by lentiviruses. Scale bar, 100 μm. Iba1 (red), CD206 (green), and DAPI (blue).

e) Quantitation of pan-macrophages (Iba1$^+$) and M2-macrophages (CD206$^+$) densities in xenografts derived from U87ΔEGFR cells with the indicated modification by lentiviruses. n = 5 biological replicates, mean ± SEM, $^{****}P < 0.0001$, ns: not significant, one-way ANOVA with Tukey's multiple comparisons test.

f) Quantitation of the fraction of M2 macrophages (CD206$^+$). The fraction was determined by M2 macrophages (CD206$^+$) among pan-macrophages (Iba1$^+$) in xenografts derived from U87ΔEGFR cells with the indicated modification

by lentiviruses. Data are represented as means ± SEM. *P < 0.05, ns: not significant, one-way ANOVA with Tukey's multiple comparisons test.

g) Kaplan–Meier (left) and log-rank P-value (right) analyses of mice bearing orthotopic xenografts of U87ΔEGFR cells with the indicated modification by lentiviruses.
